# Extracellular vesicle sorting of α-Synuclein is regulated by sumoylation

**DOI:** 10.1007/s00401-015-1408-1

**Published:** 2015-03-17

**Authors:** Marcel Kunadt, Katrin Eckermann, Anne Stuendl, Jing Gong, Belisa Russo, Katrin Strauss, Surya Rai, Sebastian Kügler, Lisandro Falomir Lockhart, Martin Schwalbe, Petranka Krumova, Luis M. A. Oliveira, Mathias Bähr, Wiebke Möbius, Johannes Levin, Armin Giese, Niels Kruse, Brit Mollenhauer, Ruth Geiss-Friedlander, Albert C. Ludolph, Axel Freischmidt, Marisa S. Feiler, Karin M. Danzer, Markus Zweckstetter, Thomas M. Jovin, Mikael Simons, Jochen H. Weishaupt, Anja Schneider

**Affiliations:** 1 Department of Psychiatry and Psychotherapy, University Medicine Göttingen, Von-Siebold-Str. 5, 37075 Göttingen, Germany; 2 Cluster of Excellence “Nanoscale Microscopy and Molecular Physiology of the Brain” (CNMPB), Göttingen, Germany; 3 Max-Planck-Institute for Experimental Medicine, Hermann-Rein-Str. 3, 37075 Göttingen, Germany; 4 Department of Neurology, University Medicine Göttingen, Robert-Koch-Str. 40, 37075 Göttingen, Germany; 5 German Center for Neurodegenerative Diseases (DZNE), Göttingen, Von-Siebold-Str. 5, 37075 Göttingen, Germany; 6 Laboratory of Cellular Dynamics, Max-Planck-Institute for Biophysical Chemistry, Am Faßberg 11, 37077 Göttingen, Germany; 7 Max-Planck-Institute for Biophysical Chemistry, Am Faßberg 11, 37077 Göttingen, Germany; 8 Department of Neurology, Ludwig-Maximilians-University Munich, Marchionistr. 15, 81377 Munich, Germany; 9 Department of Neuropathology and Prion Research, Ludwig-Maximilians-University Munich, Feodor-Lynen-Str. 23, 81377 Munich, Germany; 10 Department of Neuropathology, University Medicine Göttingen, Robert-Koch-Str. 40, 37075 Göttingen, Germany; 11 Paracelsus-Elena Klinik, Klinikstr. 16, 34128 Kassel, Germany; 12 Department of Molecular Biology, University Medicine Göttingen, Humboldtallee 23, 37073 Göttingen, Germany; 13 Department of Neurology, Ulm University, Albert-Einstein-Allee 11, 89081 Ulm, Germany; 14 Charcot Professorship for Neurodegeneration, Department of Neurology, Ulm University, Albert-Einstein-Allee 11, 89081 Ulm, Germany

**Keywords:** Exosomes, α-Synuclein, SUMOylation, Spreading

## Abstract

Extracellular α-Synuclein has been implicated in interneuronal propagation of disease pathology in Parkinson’s Disease. How α-Synuclein is released into the extracellular space is still unclear. Here, we show that α-Synuclein is present in extracellular vesicles in the central nervous system. We find that sorting of α-Synuclein in extracellular vesicles is regulated by sumoylation and that sumoylation acts as a sorting factor for targeting of both, cytosolic and transmembrane proteins, to extracellular vesicles. We provide evidence that the SUMO-dependent sorting utilizes the endosomal sorting complex required for transport (ESCRT) by interaction with phosphoinositols. Ubiquitination of cargo proteins is so far the only known determinant for ESCRT-dependent sorting into the extracellular vesicle pathway. Our study reveals a function of SUMO protein modification as a Ubiquitin-independent ESCRT sorting signal, regulating the extracellular vesicle release of α-Synuclein. We deciphered in detail the molecular mechanism which directs α-Synuclein into extracellular vesicles which is of highest relevance for the understanding of Parkinson’s disease pathogenesis and progression at the molecular level. We furthermore propose that sumo-dependent sorting constitutes a mechanism with more general implications for cell biology.

**Electronic supplementary material:**

The online version of this article (doi:10.1007/s00401-015-1408-1) contains supplementary material, which is available to authorized users.

## Introduction

Extracellular vesicles of 40–100 nm in diameter can either be derived from the multivesicular endosome (MVE) (exosomes) or shedded from the plasma membrane (microvesicles). Both are involved in the release of toxic cellular content and intercellular transfer of proteins, lipids and RNA, and are referred to jointly as extracellular vesicles (EV) [20, 26, 37, 42, 56, 58, 64, 71].

Sorting into extracellular vesicles is regulated by binding of proteins to the ESCRT complex (endosomal sorting complex required for transport) or by ESCRT-independent pathways [61]. Several of the ESCRT machinery’s four multimeric subunits bind to ubiquitinated proteins for their subsequent delivery to sites of vesicle formation and extracellular vesicle release. Hepatocyte growth factor-regulated tyrosine kinase substrate (HRS) protein, TSG101 and Vps36, which are components of ESCRT-0, -I and -II complexes, respectively, contain Ubiquitin-binding domains for recruitment of ubiquitinated membrane cargo into extracellular vesicles. However, recent data indicate that ubiquitination of membrane proteins may not be the only determinant for ESCRT interaction. ESCRT-dependent sorting to lysosomes has been reported for several non-ubiquitinated proteins [e.g. the T cell co-receptor CD4 and the delta opiod receptor (DOR)] [59]. It is not known whether this represents a truly Ubiquitin-independent protein ESCRT interaction or if these cargoes might associate with ubiquitinated interaction partners, which mediate Ubiquitin-dependent sorting to the ESCRT machinery. It therefore remains unclear whether sorting determinants exist for Ubiquitin-independent sorting into the canonical ESCRT pathway.

Posttranslational attachment of SUMO (small Ubiquitin like modifier) to proteins plays an important role in the regulation of protein–protein interactions, enabling or inhibiting protein binding [22]. Sumoylated heterogeneous nuclear ribonucleoprotein A2B1 (hnRNPA2B1) interacts with short specific miRNA motifs and controls their loading into extracellular vesicles. Interestingly, hnRNPa2B1 in extracellular vesicles is sumoylated [73].

We examined the function of posttranslational sumoylation as a possible mediator of ESCRT interaction and thereby as a sorting factor for protein release with extracellular vesicles. Here, we show that sumoylation of proteins can mediate their ESCRT-dependent sorting into extracellular vesicles. We demonstrate that SUMO is recruited to ESCRT formation sites by interaction with phosphoinositols and requires ESCRT subunits Tsg101, VPS4 and the ESCRT-associated protein Alix.

Futhermore, we provide evidence that release of the cytosolic protein α-Synuclein within extracellular vesicles is SUMO-dependent. α-Synuclein is the major constituent of intracellular pathological aggregates in Parkinson’s disease (PD) and dementia with Lewy bodies (DLB). The progression of α-Synuclein pathology in PD seems to follow a stereotypical anatomical path through the brain [9]. This, together with the emergence of aggregated α-Synuclein in transplanted embryonic nigral cells in PD patients and cell to cell transfer of α-Synuclein in mouse brain and cell culture leads to the assumption of interneuronal spreading of disease pathology [13, 14, 27, 44]. α-Synuclein can be isolated with extracellular vesicles from cell culture media [2, 16, 28, 41]. We have shown that vesicular α-Synuclein may be internalized more efficiently by recipient cells than the free protein and induce greater toxicity [12]. Therefore, transfer of α-Synuclein by extracellular vesicles might play an important role in synucleinopathies. Here, we provide evidence that α-Synuclein is present in extracellular vesicles from human cerebrospinal fluid and identify SUMO as a regulator of its sorting to the extracellular vesicle pathway and possibly interneuronal spreading of α-Synuclein pathology.

## Materials and methods

### Reagents

Primary antibodies were: mouse monoclonal antibodies against Myc clone 9E10 (Sigma), Flotillin-2 (BD Biosciences), α-Synuclein (Invitrogen), Alix (BD Biosciences), TSG101 (GeneTex Inc., Irvine, CA, USA), CD63 (BD Biosciences), 6E10 anti-APP (Signet), GAPDH clone 6C5 (Abcam), rabbit anti-Glutamate Receptor GluR2/3 (Chemicon), rabbit anti-Glutamate Receptor GluR1 (Chemicon), rabbit anti-Calnexin (StressGene), rabbit anti-GFP (Invitrogen), rabbit anti-Integrin β5 (Millipore), rabbit anti-UBC9 (Santa Cruz), and rat anti-LAMP1 (1D4B) (Santa Cruz). SUMO-2 antibody was kindly provided by F. Melchior (Heidelberg, ZMBH, Germany) [5]. Secondary antibodies were obtained from Dianova and Invitrogen.

### Plasmids and siRNA

The following plasmids were used: pEYFP-N1 (Clontech, Mountain View USA), Rab5Q79L GFP (M. Zerial, MPI-CBG, Dresden), pcDNA3 ∆N-α-Synuclein lacking the residues 2–19 was provided by H. Karube, Dept. of Neurology, Yamagata, Japan [34], pcDNA3 Myc-SUMO-2, pcDNA3 Myc-SUMO-2 ∆GG, pEYFP SUMO-1, pEYFP SUMO-1 ∆GG (a C-terminal deletion mutant that cannot be conjugated), pcDNA3 Myc-α-Synuclein, pcDNA3 Myc-α-Synuclein 2KR (bearing the double mutation K96R K102R), pcDNA3 Myc-α-Synuclein 2AA (D98A E104A) [38], pTE1E2S1 (which codes for the expression of SUMO-1 and the E1 and E2 enzymes of the sumoylation pathway [70]), pT7.7 encoding for human wild-type α-Synuclein (courtesy of the P. Lansbury laboratory, Harvard Medical School, Cambridge, MA). Fusion constructs α-Synuclein hGLuc1 (S1) and α-Synuclein hGLuc2 (S2) were generated as described previously [51]. SUMO-2-luciferase construct (SUMO-2-S3) was created by cloning the amino-terminal fragment of humanized Gaussia Luciferase including the same linker as used in S2 into *BamHI/EcoRI* sites of pcDNA3. SUMO-2 was subsequently subcloned into *EcoRI/XhoI* sites. PcDNA3 Myc-α-Synuclein-SUMO-2 ∆GG, pcDNA3 GFP-SUMO-2 ∆GG, pcDNA3 GFP-Ubiquitin ∆GG were cloned as described below. Further plasmids used were pR4 PLP-Myc, pcDNA3 MLV-Gag-GFP (Addgene Plasmid 1813, W. Mothes, Yale University School of Medicine), and GFP-VPS4 (E233Q) (P. Woodman, University of Manchester, UK). PcDNA3 GFP-SUMO-2 ∆GG SIM (with the triple mutation Q30A F31A I33A) was generated by site-directed mutagenesis according to the manufacturer’s protocol (Quick Change site-directed mutagenesis kit, Stratagene, CA, USA). pShuttleCMV GFP-APPsw was kindly provided by Patrick Keller (Max-Planck-Institute for Molecular Cell Biology and Genetics, Dresden, Germany). SUMO-2 cDNA (NM_006936.2) was amplified by PCR using the primers (5′–3′) fwTCATCAGCGGCCGCGATGTCCGAGGAGAA and rev AGCAGCAGACGGCAGCGTAGTCTAGAAAAAAA thereby eliminating the nucleotides that encode the C-terminal diglycine motif (named SUMO-2 ∆GG). SUMO-2 ∆GG was introduced 3′ terminally of GFP-APPsw via NotI and XbaI restriction sites including a linker of 15 nucleotides between APPsw and SUMO-2 ∆GG. For each fusion construct, three PCR reactions were performed. The first PCR reaction was setup for the 5′-part; the second PCR reaction for the 3′ part of the construct. The PCR products of both reactions were combined within the third PCR reaction using the outer primers only. PCR primers for the amplification of SUMO-2 (NM_006936.2) and Ubiquitin (NM_021009.5) were designed to eliminate the nucleotides that encode the C-terminal diglycine motif (named SUMO-2 ∆GG and Ubiquitin ∆GG) and to create a short linker between both constructs that were fused. The product of the third PCR reaction was cloned via *Hind*III and *Xho*I restriction sites into the pcDNA3 vector (Life Technologies, Darmstadt, Germany). pcDNA 3 Myc-SUMO2 cleft: SUMO2 cDNA with deletion of the C-terminal GG and the mutations Q30A, F31A, K32A, I33A, L42A, and Y46A was cloned into pcDNA 3 Myc vector via BamHI and XhoI restriction sites. SUMO2 cDNA with deletions of the C-terminal GG and the mutations H16A, Q30A, F31A, K32A, I33A, H36A, L42A, Y46A, and D62A (cleft + loop) and Q30A,F31A,K32A,I33A,L42A,Y46A (cleft) were cloned into pcDNA 3 Myc vector via BamHI and XhoI restriction sites. The following primer pairs (5′–3′) were used for the various constructs: GFP-Ubiquitin ∆GG: GFP fw ACCCAAGCTTATGGTGAGCAA, rev ATGGACGAGCTGTACAAGGCAGCGATGCAGATCTT; Ubiquitin fw TACAAGGCAGCGATGCAGATCTTCGTGAA, rev ACCTGGTCCTTCGTCTCAGAGCAGCGTAGCTCGAGTTT; GFP-SUMO-2 ∆GG: GFP fw ACCCAAGCTTATGGTGAGCAA, rev ATGGACGAGCTGTACAAGGCAGCGATGTCCGA; SUMO-2 fw AGCTGTACAAGGCAGCGATGTCCGAGGAGAAGCCC, rev AGCAGCAGACGGCAGCGTAGCTCGAGAAAA; α-Synuclein-SUMO-2 ∆GG: syn fw ATCTGAAGCTTATGGATGTATTCAT, rev TACGAACCTGAAGCCGCAGCGATGTCCGA; SUMO-2: fw AAGCCGCAGCGATGTCCGAGGAGAAGCCC, rev AGACGGCAGCGTAGCTCGAGAAA. The following siRNAs from Qiagen GmbH, Germany, were used: TSG101: sense sequence 5′-CUGUAUAAACAGAUUCUAAdTdT-3′, antisense sequence 5′-UUAGAAUCUGUUUAUACAGdTdT-3′; Alix: sense sequence 5′-GAACCUGGAUAAUGAUGAAdTdT-3′, antisense sequence 5′- UUCAUCAUUAUCCAGGUUCdTdT-3′. The UBC9 siRNA from Dharmacon was CAAAAAAUCCCGAUGGCACUU sense sequence, GUGCCAUCGGGAUUUUUUGUU antisense sequence.

### Cell culture, transfection and siRNA delivery

The oligodendroglial cell line Oli-neu was provided by J. Trotter, University of Mainz, Germany, and cultured as described [19]. Mouse neuroblastoma N2a cells were maintained as described in [55]. Both, Oli-neu and N2a cells were plated on glass coverslips or 10-cm plastic dishes and transfected with TransIT (Mirus Bio LLC, Madison, WI, USA) according to the manufacturer’s protocol. siRNA was delivered to N2a cells by Oligofectamine (Invitrogen) and cells were transfected 36 h later with the different plasmids, followed by medium exchange after 8 h and collection of extracellular vesicles. As a control, cells were mock transfected with oligofectamine reagent in the absence of siRNA.

Primary cortical neurons were prepared from E16 NMRI mouse embryos and cultured on poly-lysine-coated glass coverslips or plastic dishes in serum-free MEM supplemented with B27 (Invitrogen). Infection with adenovirus-associated virus (AAV) 6 encoding either human α-Synuclein wild type or α-Synuclein 2KR was performed between days 3 and 4 in vitro. Medium was changed on day 6 and extracellular vesicles were prepared on day 7, 16 h after medium exchange.

### Luciferase activity assay

Luciferase activity assay in extracellular vesicles α-Synuclein and SUMO-2-luciferase constructs [α-Synuclein fused to full-length gaussia luciferase (syn phGluc); C- or N-terminal fragments of split phGluc fused to α-Synuclein (syn-S2) or SUMO-2 (SUMO-2-S3)] were transfected into HEK293 cells in 10-cm dishes. 16 h after transfection, cells were washed with PBS and replaced with serum- and phenol-red free media. After 48 h, medium was collected and extracellular vesicles were prepared as described below. Cells were washed with PBS and lysed in PBS using sonication. Luciferase activity from protein complementation was measured using same amounts of total protein of cell lysates and extracellular vesicle fractions in an automated plate reader at 480 nm following the addition of the cell permeable substrate, coelenterazine (40 μM; PJK GmbH, Kleinbittersdorf, Germany) with a signal integration time of 2 s.

### Immunofluorescence stainings

Immunofluorescence stainings were performed according to standard protocols. Images were taken with a laser scanning microscope (Leica SP2, Leica, Mannheim, Germany) with a 63 oil plan-apochromat objective.

### Virus production

Virus production rAAV6 were used to express α-Synuclein wild type and α-Synuclein 2KR (K96R and K102R) under the neuron-specific human Synapsin 1 (hSyn1) promotor [40]. Virus production was performed as described [38]. In brief, vectors were propagated in HEK 293 T cells together with the pDP6 helper plasmid. Virus particles were purified by iodixanol step purification, followed by heparin affinity chromatography with 1 ml HiTrap Heparin QFF columns (GE Healthcare). Virus particles were desalted by dialysis against PBS and titrated by quantitative PCR.

### Purification of extracellular vesicles

Extracellular vesicles were isolated as described previously [65]. Cells were grown on plastic dishes. 8 h after transfection, cells were washed three times in phosphate-buffered saline (PBS) and incubated in serum-free DMEM for 16 h. Culture medium was then collected and the supernatants were subjected to subsequent centrifugation steps performed at 4 °C: 3500×*g* 10 min, 2 times 4500×*g* for 10 min, 10,000×*g* for 30 min and 100,000×*g* with a TLA 100.3 rotor (Beckman-Coulter, *k*-factor 60.6) for 60 min. The 100,000×*g* pellet was washed once with PBS before resuspension in sample buffer. To quantify extracellular vesicle release, protein parent cell lysates were scraped into 1 % CHAPS, 5 mM EDTA, 50 mM Tris–HCl, pH 8.0 lysis buffer and extracellular vesicle fractions as well as postnuclear supernatants of the cell lysates were subjected to Western blotting. The ratio of extracellular vesicle protein versus cellular protein levels was calculated after scanning the blots followed by Image J analysis.

Extracellular vesicles from human cerebrospinal fluid for Western blot analysis were prepared from 5 ml of cerebrospinal fluid after the written informed consent was given. Analysis of patient cerebrospinal fluid was approved by the ethical committee of the Medical Faculty, University Medicine Goettingen (IRB 02/05/09).

### Sucrose gradient ultracentrifugation

A 100,000×*g* pellet containing extracellular vesicles was prepared as described above and resuspended in 400 μl of 0.25 M sucrose in 10 mM HEPES, pH 7.4 and layered on top of the discontinuous sucrose density gradient consisting of 8 fractions from 0.25 to 2.5 M sucrose. The gradient was centrifuged for 16 h at 200,000×*g* with a 60Ti rotor (Beckman-Coulter, *k*-factor 80.1) or SW41 Ti rotor (Beckman-Coulter, *k*-factor 124), and fractions were recovered and centrifuged at 100,000×*g* after dilution in PBS for 1 h, followed by resuspension of the resulting pellet in sample buffer and Western blot analysis.

### Nanoparticle tracking analysis (NTA)

Cell culture supernatants were centrifuged at 3500×*g* to remove cellular debris. The supernatant was diluted 1:1 in PBS (Gibco) and subjected to a NanoSight LM14 instrument equipped with a 532-nm laser (NanoSight Ltd., Amesbury, UK). Samples were measured in triplicates for 30 s. Particle numbers were analysed with the Nanoparticle Tracking Analysis (NTA) 2.3 software.

### Single particle fluorescence assay for vesicle-binding properties of sumoylated and non-sumoylated α-Synuclein

Expression and purification of α-Synuclein and sumoylated α-Synuclein was performed as described previously [38]. Fluorescent labelling with Alexa Fluor-647-O-succinimidylester (Molecular Probes, USA) was performed as described [23]. Green-labelled small unilamellar dipalmitoyl-sn-glycero-3-phospho-choline lipid vesicles (DPPC-SUV) were generated as described [31]. Fluorescence correlation spectroscopy (FCS) and scanning for intensely fluorescent targets (SIFTs) measurements for the quantification of α-Synuclein vesicle binding were carried out on an Insight Reader (Evotec-Technologies) with dual colour excitation at 488 and 633 nm as described before [31]. All measurements were performed after incubation of DPPC-SUV with labelled α-Synuclein for at least 30 min. Measurements under equilibrium conditions were performed >2 h after addition of unlabelled non-sumoylated α-Synuclein. Data from at least three parallel samples were recorded for each experimental group.

### Electron microscopy

Extracellular vesicles were prepared from cerebrospinal fluid and culture medium as described above. The 100,000×*g* pellet was fixed with 4 % paraformaldehyde and was adsorbed to glow-discharged Formvar-carbon-coated copper grids by floating the grid for 10 min on 5 μl droplets on Parafilm. The grids were negatively stained with 2 % uranyl acetate containing 0.7 M oxalate, pH 7.0, and imaged with a LEO EM912 Omega electron microscope (Zeiss, Oberkochen). Digital micrographs were obtained with an on-axis 2048 CCD camera (Proscan, Scheuring).

### Membrane preparation

Cells were washed twice with ice-cold PBS and collected into 200 µl homogenization buffer (20 mM Na-HEPES, 1 mM EDTA, 0.32 M sucrose, pH 7.0). The cells were mechanically disrupted by 10× pipetting up and down through a yellow pipette tip and finally 10× through a 27G needle. Cells were centrifuged at 4000 rpm for 5 min at 4 °C. The postnuclear supernatant was then ultracentrifuged with 196,000×*g* for 30 min at 4 °C. The pellet containing membrane fraction and cytosol were resolved in sample buffer and subjected to SDS-PAGE and Western blotting.

### Electrochemiluminescence assay for α-Synuclein quantification

Quantification of α-Synuclein protein in cell lysates and extracellular vesicles derived from primary neuron cultures was performed as described [39] with slight modifications. Standard 96-well Multi-Array plates (Meso Scale Discovery, Gaithersburg, USA) were coated with 3 µg/ml antibody MJF-1 clone 12.1 (kindly provided by Dr. Liyu Wu, Epitomics, Burlingame, USA) in PBS. Plates were incubated overnight at 4 °C without shaking. All further steps were performed at room temperature. After washing, the plates three times with 150 µl PBS + 0.05 % Tween-20 blocking was performed with 150 µg 1 % BSA (Meso Scale Discovery) for one hour under shaking at 300 rpm. A standard curve of recombinant α-Synuclein (kindly provided by Dr. Omar el-Agnaf, United Arab Emirates University, Al Ain, United Arab Emirates) was prepared in serial fourfold dilution starting at 25,000 pg/ml. After washing as above, standards and samples were applied at 25 µl per well in duplicate. Binding of analytes was allowed for 1 h under shaking at 700 rpm. Washing was done as above followed by addition of 25 µl Sulfo-TAG labelled anti-α-Synuclein clone 42/α-Synuclein (BD Transduction Laboratories, Heidelberg, Germany) at 1 µg/ml. Incubation was done for 1 h under shaking at 700 rpm. After washing again 150 µl 2 × Read Buffer T (Meso Scale Discovery™) was applied to each well and plates were measured in a Sector Imager 6000 (Meso Scale Discovery™). Data analysis was performed using MSD Discovery Workbench 3.0 Data Analysis Toolbox.

### Expression and purification of sumoylated α-Synuclein

The expression and purification procedure of human sumoylated wild-type α-Synuclein was based on [38]. In brief, BL21 competent *E. coli* cells were co-transformed with the tricistronic plasmid pTE1E2S1, which codes for the expression of SUMO-1 and the E1 and E2 enzymes of the sumoylation pathway [70], and the pT7.7 encoding for human wild-type α-Synuclein (courtesy of the P. Lansbury laboratory, Harvard Medical School, Cambridge, MA). After enzymatic degradation of DNA, the bacterial extracts were heat precipitated at 95 °C for 10 min and the supernatant subjected to column chromatography (GE Healthcare Äkta system) with a sequence of 3 columns: Q Shepharose fast flow, HiLoad 26/600 Superdex 200, and Mono Q 4.6/100 PE. Fractions of sumoylated α-Synuclein were combined and concentrated with an Amicon Ultracel Filter (10 kDa, Millipore), and purity assessed by polyacrylamide gel electrophoresis (PAGE) and electrospray ionization mass spectroscopy (ESI–MS). The protein concentration was estimated using a molar extinction coefficient at 280 nm of 9080 M^−1^ cm^−1^.

### Membrane binding of SUMO-2: titration of SUMO-2-MFM with SUVs

The single Cys52 of SUMO-2 was labelled with the ESIPT probe MFM [60]. Unreacted Cys residues (<15 %) were blocked with a tenfold excess of *N*-ethylmaleimide and the labelled protein was purified through a PD10 column to separate it from unreacted probe. Single Unilamellar Vesicles (SUVs) were prepared by sonication as described previously [18] with a composition based on mixtures of POPC, POPS, and PIPs: (Avanti Polar Lipids, Alabaster, Alabama, USA). The relative molar compositions and approximate net relative molar charge densities were as follows (other ratios were also employed): POPC, 100, 0); POPC:POPS, 90:10; −0.1]; POPC:POPS:PI(3)P 85:10:5; −0.13]; POPC:POPS:PI(5)P 85:10:5; −0.14); POPC:POPS:PI(3,5)P2 85:10:5; −0.2]; POPC:POPS:PI(4,5)P2 85:10:5, −0.2; and POPC:POPS:PI(3,4,5)P3, 85:10:5, −0.25. SUV stocks were quantified by determining inorganic phosphorus [24] and vesicles were used within 10 days of their preparation.

The titrations of the labelled protein with SUVs were performed with a new microplate assay (slopes, to be published elsewhere; for more details contact author TMJ). This assay avoids the problems associated with conventional fluorescence assays based on the addition of lipids to protein: signal contributions from lipid (emission and scattering), and photobleaching during the course of prolonged (and tedious) sequential additions. The strategy of slopes exploits the maximal sensitivity of a titration performed with concentrations of the reagent in excess (the lipid) varied around the anticipated value(s) of the dissociation constant *K*_d_s. Lipid mixtures of various concentrations bracketing the estimated *K*_d_ are prepared with a small number of protein concentrations in the sub-µM range. Measurements of protein-derived signals at constant lipid concentration and varying protein yield straight linear relationships, the slopes of which depend on the fraction *α* of bound protein, given by the quantity *α* = [lipid]/(*K*_d_ + [lipid]). The slopes measured for a small number of protein concentrations are plotted versus the lipid concentrations (usually 4, including 0 concentration), from which *K*_d_ and the fluorescence enhancement factor are calculated from the relation: slope = *f*0[1 + (fe − 1)α, where *f*0 is the slope corresponding to 0 lipid concentration and fe is the (enhanced) fluorescence of the bound protein relative to that of the free protein. Important advantages of this method are: (1) parallel readout in a microplate reader; (2) bottom readout with small optical path length and thus minimal scattering artefacts; (3) minimal reagent requirements; the protein concentration can be reduced to a minimum dictated only by the background signal and the probe sensitivity; (4) lack of photobleaching artefacts (single endpoint determinations); and (5) accurate correction for potential signals introduced by components of the lipid mixtures. In the experiments reported in this study, the slopes method was enhanced by considering the apparent (measured) *K*_d_ value as the inverse of the sum of the reciprocal individual *K*_d_s for each lipid component weighted by the respective molar fractions. Thus, the entire series of lipid mixtures could be subjected to a global fitting procedure such as to derive the individual *K*_d_ values for POPC, POPS, and the PIs (those with affinities greater than that of POPS). The fe values were allowed to vary with lipid composition. We note that the derived *K*_d_ values implictly contain (as a multiplicative factor) the binding stoichiometry, i.e. the number of lipid molecules participating in the formation of the protein–lipid complex.

Solutions of SUMO-2-MFM (100, 200 and 300 nM) were prepared in 25 mM Na-HEPES, 100 mM KCl, pH 7.26, with different (usually 7) SUV concentrations up to 120 µM. Replicates (usually 2) of 100 µl were introduced into a 96-well quartz microplate (Hellma Analytics, Germany) exhibiting very low background with excitation in the near-UV. After at least 10 min of incubation at room temperature, the MFM T-band fluorescence excited at 340 nm was recorded at >510 nm in a Pherastar plate reader (BMG Labtech, Germany) operated in bottom readout, well scan mode with a matrix of 10 × 10, a scan diameter of 5 mm, and 20 flashes per well. Samples without protein and/or lipid were included to establish blank values and the lipid contributions to the measured signals. The data were analysed with procedures implemented in Mathematica (Wolfram Research).

### Expression of recombinant SUMO-2 for NMR

SUMO-2 was cloned into pET11 and expressed as previously described [52]. For N^15^ labelling of SUMO-2 proteins, bacterial cells were grown in one litre LB at 37 °C until the culture reached an optic density (OD_600_) of 0.6. Bacteria cultures were then centrifuged and resuspended in 500 ml standard Minimal M9 media containing 3 g glucose. Following 30 min of incubation, 1 g N^15^H_4_Cl was added to the medium, and cells were further grown for one hour at 37 °C, before induction with 1 mM IPTG. SUMO purification was performed as described, except that for gel-filtration analysis a buffer containing 20 mM NaH_2_PO_4_/Na_2_HPO_4_ pH 6.8, 100 mM KCl, 2 mM DTT was used.

### NMR spectroscopy

To study the membrane binding of SUMO-2 by NMR 200 µM ^15^N-labelled SUMO-2 in 20 mM NaH_2_PO_4_/Na_2_HPO_4_, pH 6.8, 100 mM KCl, 1 mM DTT was titrated with increasing concentrations of 8, 16 and 32 mM DHPC (1,2-dihexanoyl-sn-glycero-3-phosphocholine). ^1^H,^15^N-HSQC spectra were acquired at 600 MHz and 22 °C on a triple resonance room temperature probe with 16 transients, 2048 × 256 total points and sweep widths of 8418 × 2129 Hz (^1^H × ^15^N). Carrier frequencies were set to the water resonance for ^1^H and to 117 ppm for ^15^N. Resonance assignments were taken from BMRB entry 11267. The normalized weighted average chemical shift difference for the amide proton and nitrogen was calculated according to $\Delta \delta \, \left( {\text{HN}} \right) = [\Delta \delta_{\text{H}}^{ 2} + (0. 2 \; \times \;\Delta \delta_{\text{N}} )^{ 2} ]^{ 1/ 2} .$

## Results

### Sumoylation is a sorting signal for release within extracellular vesicles

**Fig. 1:**
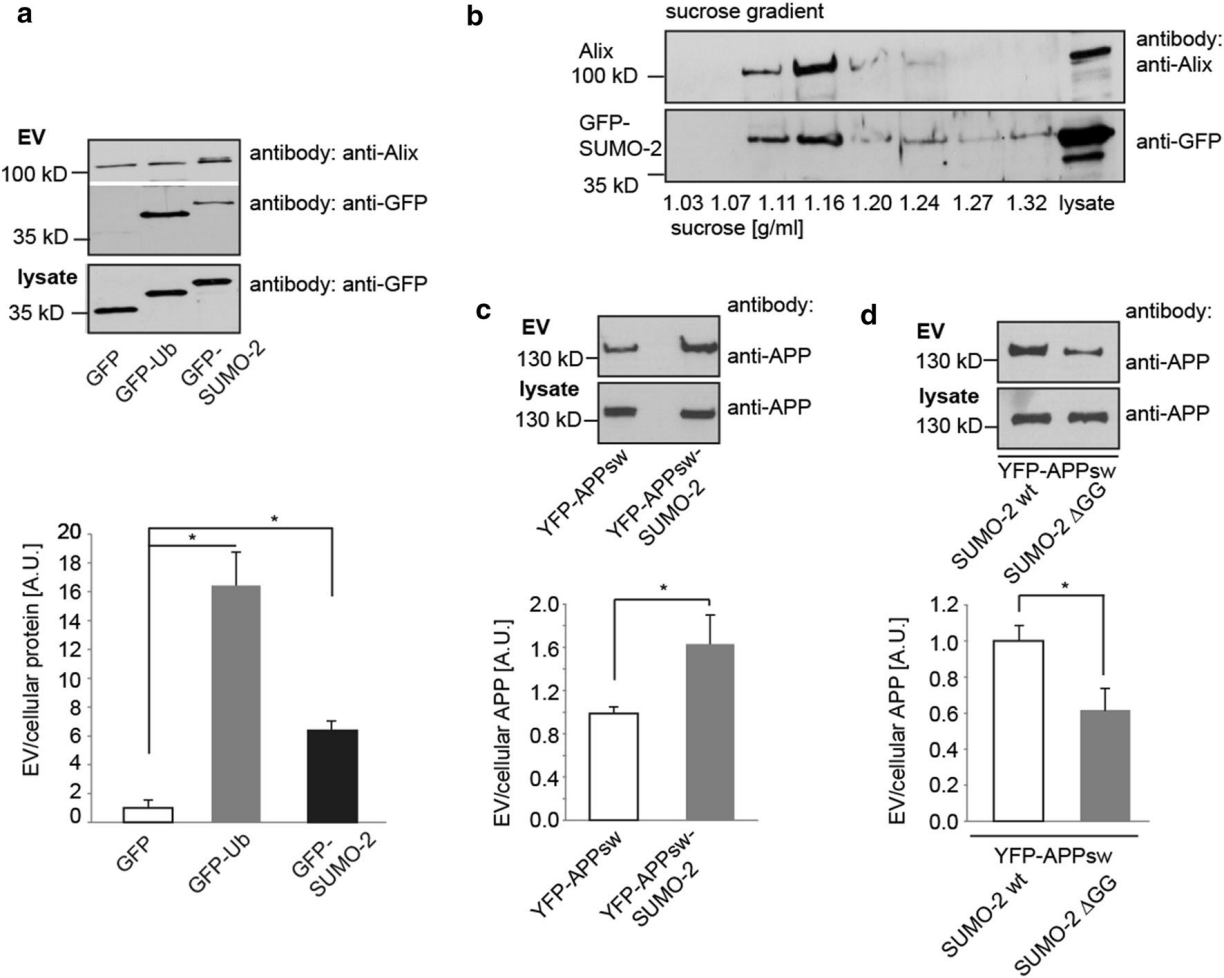
SUMO-2 is released with extracellular vesicles. **a** N2a cells were transiently transfected with GFP or GFP either fused to a conjugation-deficient Ubiquitin mutant GFP-Ub or to the conjugation-deficient SUMO-2 mutant GFP-SUMO-2. Lysates and EV fractions were subjected to Western blot analysis to detect GFP, GFP-Ub and GFP-SUMO-2 in the cell lysate (lys) and the EV-fraction. The blot was also probed for Alix as a marker for EVs in the different preparations. Blots were scanned and analysed with Image J to determine the ratio of extracellular vesicle (EV) to cellular protein. Results are given as means + SEM, *p < 0.05, two-side *t* test (*n* = 8). **b** The 100,000*g* pellet from GFP-SUMO-2 transfected N2a cells was subjected to a discontinuous sucrose gradient. Fractions from 1.03 to 1.32 g/ml sucrose were blotted with antibodies against Alix and GFP. SUMO-2 was recovered from 1.11 to 1.16 g/ml fractions, similar to Alix

To elucidate if sumoylation may act as a sorting signal for ESCRT-dependent release within extracellular vesicles we generated a green fluorescent protein (GFP)-SUMO-2 ∆GG fusion construct, in which the deletion of the two C-terminal glycine residues prevents conjugation of SUMO to proteins. If not otherwise stated, conjugation-deficient mutants of SUMO-2 were for all further experiments. We prepared extracellular vesicles according to previously described protocols by subsequent centrifugation rounds including a final 100,000×*g* ultracentrifugation step from the medium of mouse neuroblastoma cells (N2a) as previously described [65]. N2a cells were transfected with either GFP-SUMO-2 or GFP as a negative control. Whereas GFP was excluded from the extracellular vesicle fraction, GFP-SUMO-2 was sorted into extracellular vesicles. The same was observed for GFP fused to a conjugation-deficient-Ubiquitin construct (GFP-Ub) that we chose as a positive control based on the fact that mono-ubiquitination is a well-established sorting factor for release within extracellular vesicles (Fig. 1a). On a sucrose gradient, GFP-SUMO-2 banded at a density of 1.11–1.16 g/ml similar to the extracellular vesicle marker protein Alix (Fig. 1b). To rule out unspecific sorting of GFP-SUMO-2 into extracellular vesicles mediated by the GFP-fusion protein we prepared extracellular vesicles from N2a cells transiently transfected with either GFP-SUMO-2 or Myc-SUMO-2. Sorting of GFP- and Myc-tagged SUMO-2 into extracellular vesicles (Supplementary Fig. S1a) was indistinguishable, ruling out sorting mediated by GFP.

SUMO-1 and SUMO-2 share a 47 % homology [47]. In comparison of the GFP-fusion constructs of both SUMO isoforms, SUMO-1 was retrieved from the extracellular vesicle fraction, albeit to a lesser extent than SUMO-2 (Supplementary Fig. S1b).

Having shown that SUMO protein is released within extracellular vesicles, we next asked whether it serves as a sorting signal. While extracellular vesicle targeting signals had not been described for cytosolic proteins before, mono-ubiquitination of transmembrane proteins was reported to mediate their sorting to extracellular vesicles [54]. We therefore explored whether sumoylation might also target transmembrane proteins into extracellular vesicles. The amyloid precursor protein APP is a class 1 transmembrane protein involved in the pathogenesis of Alzheimer’s Disease. We transfected YFP-APP bearing the Swedish mutation (APPsw K670N M671L) or the corresponding C-terminal SUMO-2 fusion construct YFP-APPsw-SUMO-2 into N2a cells. The fusion of conjugation-deficient SUMO2 to YFP-APP (YFP-APP-SUMO-2) significantly increased release of APP with extracellular vesicles (Fig. 1c, Supplementary Fig. S1c). Likewise, co-expression of APPsw with the conjugation-competent SUMO-2 wild type resulted in increased full-length APP release when compared to co-transfection of APPsw with the conjugation-deficient mutant SUMO-2 (Fig. 1d). Total amounts of EVs in both conditions were quantified by nanoparticle tracking analysis and did not show significant differences between cells transfected with YFP-APPsw and YFP-APPsw-SUMO-2 or cells co-tranfected with APPsw and SUMO-2 wild type or with the conjugation-deficient SUMO-2 ∆GG (Suppl. Table S1).

### Extracellular vesicle release of SUMO-2 is ESCRT-dependent

**Fig. 2:**
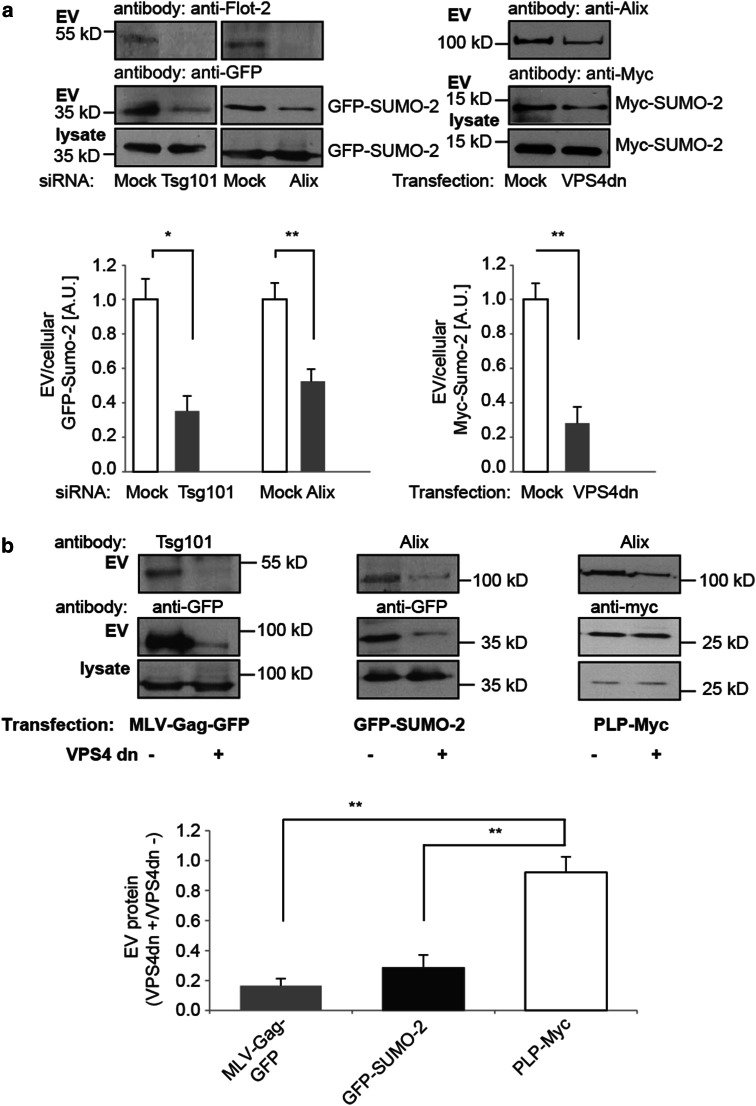
Release of SUMO-2 with extracellular vesicles is dependent on ESCRT. **a** N2a cells were treated with Tsg101 or Alix siRNA as indicated 36 h prior to transfection with GFP-SUMO-2. In the case of VPS4dn, Myc-SUMO-2 was co-transfected with the dominant negative VPS4 mutant E233Q. The ratio of extracellular vesicle (EV) to cellular SUMO-2 (lys) was determined by Western blot analysis. Results are given as means + SEM, *p < 0.05, **p < 0.005, two-side *t* test (*n* = 12 for Alix, *n* = 6 for Tsg101, *n* = 4 for VPS4dn). **b** Interference with VPS4 function inhibits release of SUMO-2 with extracellular vesicles. N2a cells were co-transfected with VPS4dn and either MLV-Gag-GFP or GFP-SUMO-2 or PLP-Myc. Cells transfected with MLV-Gag-GFP, GFP-SUMO-2 or PLP-Myc alone served as controls. Extracellular vesicles were prepared and cell lysates and vesicle pellets were subjected to Western blotting and probed with anti-GFP and anti-Myc antibodies. The ratio of protein in the extracellular vesicle fraction from cells co-transfected with VPS4dn to mock-transfected cells was quantified by Western blot analysis. Results are given as means + SEM, **p < 0.005, two-side *t* test (*n* = 5)

The biogenesis of exosomes requires inward budding of the multivesicular endosome limiting membrane to generate intraluminal vesicles which are subsequently released as extracellular vesicles upon fusion of the multivesicular endosome with the plasma membrane. In contrast, microvesicles are formed by outward budding from the plasma membrane. Proteins can be targeted to intraluminal vesicle or microvesicle budding sites by ESCRT-dependent or -independent mechanisms. To determine whether SUMO-2 is targeted to extracellular vesicles by the ESCRT machinery we used RNA interference (RNAi) against the ESCRT complex proteins Tsg101 and Alix or co-expressed the dominant negative (dn) mutant of VPS4 E233Q (See Supplementary Fig. S2 for quantification of RNAi). Depletion of either ESCRT component resulted in a marked reduction of SUMO-2 release with extracellular vesicles (Fig. 2a). Co-expression of the dominant negative VPS4 E233Q mutant together with the Moloney murine leukaemia virus Gag fused to GFP (MLV-Gag-GFP) or SUMO-2 inhibited their extracellular vesicle release while secretion of the proteolipid protein (PLP) with extracellular vesicles was not affected, consistent with previous observations that PLP is sorted into extracellular vesicles in an ESCRT-independent manner [68] (Fig. 2b).

### Interaction of SUMO-2 with the ESCRT complex does not depend on the SUMO SIM interaction motif

**Fig. 3:**
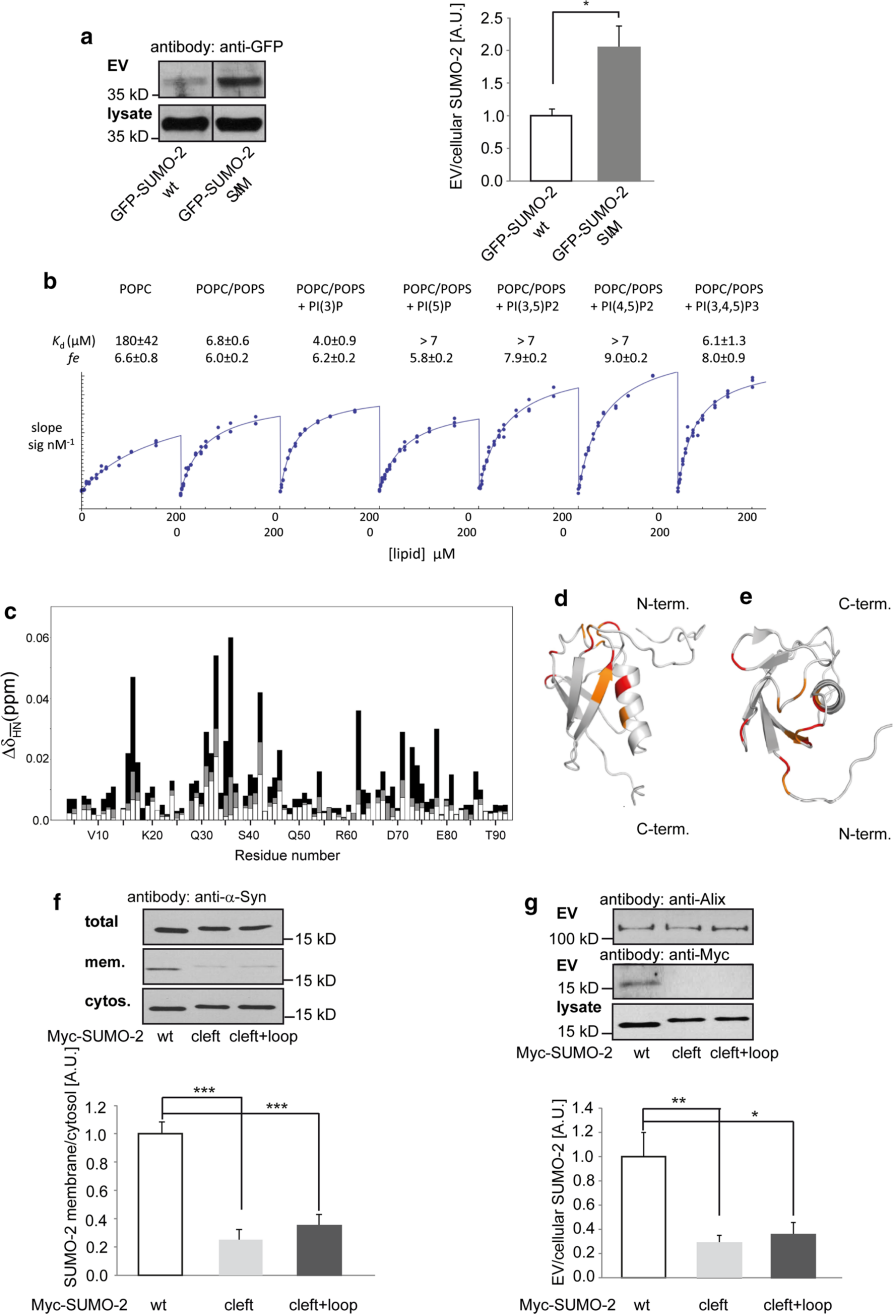
**a** Mutation of the SIM interacting motif in GFP-SUMO-2 (Q 30 F 31 I 33 to A30 A31 A 33) does not inhibit EMV SUMO-2 release. N2a cells were transfected with either SUMO-2 or the SUMO-2 triple A mutant (∆SIM). EMV release was quantified by Western blot analysis. Results are given as means + SEM, *p < 0.05, two-side *t* test (*n* = 10). **b** Titration of SUMO-2-MFM with SUVs containing POPC, POPC/POPS, or POPC/POPS supplemented with either PI(3)P, PI(5)P, PI(3,5)P2, PI(4,5)P2 or PI(3,4,5)P3. The affinities of each lipid contributing to the apparent affinity of the protein for the liposome were calculated as described in Methods. *K*_d_s are given ±standard measurement errors. The values corresponding to PI(5)P, PI(3,5)P2 and PI(4,5)P2 were too high to be determined (affinity less than that of the POPS co-lipid, i.e. >7 µM). *N* = 19 titrations; statistically significant differences were obtained for POPC versus POPS (*p* < 0.001), and POPS versus PI(3)P (*p* = 0.01). No significant difference was found for PI(3,4,5)P3 versus PS. The fluorescence enhancement factors (fe) are indicated with their respective standard measurement errors. See “Materials and methods” for further details. Membrane binding of SUMO-2 analysed by NMR spectroscopy. **c** Mean weighted ^1^H-^15^N chemical shifts of Sumo-2 at DHPC concentrations of 8 mM (*white bars*), 16 mM (*grey bars*) and 32 mM (*black bars*). Below the critical micellar concentration (CMC) of DHPC of 16 mM only few chemical shift changes in SUMO-2 were observed (**d** + **e**) The chemical shift perturbation at 32 mM DHPC is plotted onto the SUMO-2 NMR structure (pdb-code: 2AWT). Residues in *red* display a perturbation greater than 0.03 ppm and residues in orange between 0.02 and 0.03 ppm. The structure in panel d is rotated by 90º relative to panel (**e**). **f** Mutations of the SUMO-2 cleft and loop domains decrease membrane binding N2a cells transfected with Myc-SUMO-2, Myc-SUMO-2 cleft mutant or Myc-SUMO-2 cleft + loop mutant. The postnuclear supernatant of the mechanically disrupted cells was centrifuged at 196,000×*g* for 30 min to separate the membrane-containing pellet and the cytosolic supernatant. Membrane pellets and a proportion of the total cell lysate and the cytosol-containing supernatant were subjected to Western blot analysis with anti-Myc antibody to quantify the ratio of membrane associated SUMO-2. Results are given as means + SEM, ***p < 0.0005, two-side *t* test (*n* = 8). **g** Mutations of the SUMO-2 cleft and loop domains decrease extracellular vesicle release. N2a cells were transfected with Myc-SUMO-2, Myc-SUMO-2 cleft mutant or Myc-SUMO-2 cleft + loop mutant. Lysates and EV fractions were subjected to Western blot analysis to detect the different SUMO constructs in the cell lysate (lys) and the EV-fraction. The blot was also probed for Alix as a marker for EVs in the different preparations. Blots were scanned and analysed with Image J to determine the ratio of extracellular vesicle (EV) to cellular protein. Results are given as means + SEM, *p < 0.05, **p < 0.005, two-side *t* test (*n* = 9)

Having established an ESCRT-dependent mechanism for SUMO-2 release with extracellular vesicles, we next asked how SUMO-2 interacts with the ESCRT machinery. Non-covalent protein binding of SUMO is often mediated by a conserved SUMO-interaction motif (SIM) in SUMO-binding proteins, which is defined by a hydrophobic core often flanked by acidic residues [22]. The corresponding SIM interaction domain in SUMO-2 has been mapped to a groove between the α-helix and β-sheet of SUMO-2, most prominently to amino acids Q 30 F 31 I 33 [29, 66]. Mutation of these residues to alanine was shown to abolish SUMO-2 interaction with SIM domains [48, 75]. Tsg101 contains a putative SIM domain that might mediate ESCRT-dependent sorting of SUMO-2 to extracellular vesicles. To explore whether extracellular vesicle sorting of SUMO-2 is mediated by a SIM-dependent direct or indirect protein–protein interaction we introduced the QFI to AAA triple mutation into Myc-SUMO-2. Surprisingly, this mutation did not inhibit but instead increased the sorting of mutant SUMO-2 into extracellular vesicles (Fig. 3a). We therefore concluded that release of SUMO-2 with extracellular vesicles was not likely to occur via a classical SIM-mediated protein–SUMO interaction. Moreover, the disruption of the SIM interaction motif might rather increase the amount of unbound, cytosolic SUMO-2 that is available for allocation to the ESCRT formation sites.

### SUMO-2 interacts with phosphoinositols

Since the sorting of SUMO-2 into extracellular vesicles does not appear to depend on SIM-mediated protein interactions, we asked whether SUMO-2 might interact with lipids for extracellular vesicle sorting and release. In different SUMO-2 interacting proteins, such as the E3 SUMO ligase PIAS1, the tumour suppressor protein PML and the extracellular vesicle protein PMSCL1, phosphorylation of serine residues flanking the hydrophobic SIM core is a prerequisite for SUMO binding, indicating that SUMO-2 interacts with negatively charged domains [63]. Phosphorylated inositols have been demonstrated to serve as regulators of cargo sorting to the ESCRT complex and into multivesicular endosomes. To gain insight into the possible direct interaction of SUMO-2 with lipids, the recognition specificity of SUMO-2 for membranes of particular composition and charge was determined. We performed titration of the protein labelled with the polarity-sensitive excited-state intramolecular proton transfer (ESIPT) probe MFM [60] with small unilamellar vesicles according to a microplate assay devised for this purpose (see “Materials and methods”). Small unilamellar vesicles were prepared from mixtures of 1-palmitoyl,2-oleoyl-sn-glycero-3-phosphocholine (POPC) in various combinations with negatively charged lipids: phosphatidylserine (POPS, 10 %) and low fraction (5 %) of the phosphoinositides PI(3)P, PI(5)P, PI(3,5)P2, PI(4,5)P2 or PI(3,4,5)P3. The individual affinity of each lipid for SUMO-2 in the mixture was calculated from a global analysis of the combined data (Fig. 3b). We found that SUMO-2 binds to liposomes with the highest affinity for PI(3)P (*K*_d_ ~ 4.0 µM) followed by PI(3,4,5)P3 and POPS (*K*_d_ 6–7 µM). Binding to uncharged POPC was much weaker, while PI(5P, PI(3,5)P2 and PI(4,5)P2 did not exhibit affinities discernably greater than that of POPS. Thus, negatively charged lipids appear to be required for finite binding and it is possible that only one or a few PI molecules, preferentially PI(3)P, suffice for significantly increasing affinity and, thereby, specificity of SUMO binding to lipid membranes. The comparison between PI(3)P (strong binding) and PI(5)P (weak binding) indicates that electrostatic interactions do not predominate, as suggested previously [25, 32]. However, the fluorescence enhancement of the probe (fe) increased with negative charge density (Fig. 3b), indicating a strong influence of the lipid interaction site on the microenvironment of the bound protein.

Binding of SUMO-2 to either PI(3)P or PI(3,4,5)P3, which are known to recruit the ESCRT complex, might thus bring SUMO-2-modified proteins in close proximity to the ESCRT complex and explain SUMO-2-directed sorting into extracellular vesicles. Whereas PI(3)P is predominantly localized to endosomal membranes, PI(3,4,5)P3 and phosphatidylserine are enriched in the inner leaflet of the plasma membrane [30]. To distinguish between multivesicular body-mediated release and plasma membrane shedding of vesicles, we determined whether SUMO-2 was localized in intraluminal vesicles of multivesicular endosomes. To this end, we co-expressed SUMO-2 together with guanosine triphosphatase-deficient Rab5 (Rab5Q79L) which results in enlarged endosomes filled with intraluminal vesicles [3, 68]. We found that SUMO-2 was completely absent from intraluminal vesicles in contrast to PLP, which was used as a positive control for multivesicular body-mediated sorting and release (Supplementary Fig. S3a) [68]. These findings suggest that SUMO-dependent budding into extracellular vesicles might occur from the plasma membrane rather than from endosomal compartments. However, since small shedding microvesicles and multivesicular body-derived exosomes cannot be distinguished by their physical or biochemical properties, further experiments are needed to ultimately distinguish between both pathways.

### The membrane interaction motif of SUMO-2 maps to the hydrophobic cleft and nearby loops

To identify the membrane interaction motif in SUMO-2, we performed NMR spectroscopy on recombinant SUMO-2 upon 1,2-dihexanoyl-sn-glycero-3-phosphocholine (DHPC) binding (Fig. 3c–e). Below the critical micellar concentration of DHPC only few chemical shift changes in SUMO-2 were observed (Fig. 3c). Primarily residues in the hydrophobic cleft between the second β-strand and the α-helix (F31, K32, I33, L42 and Y46) showed a small chemical shift perturbation suggesting a hydrophobic interaction of the free phospholipids and the hydrophobic cleft. Above the critical micellar concentration, additional residues located to the loops at the N-terminal side of SUMO-2 (Fig. 3d + e), in particular H16, H36 and D62, displayed strong changes in chemical shifts, indicating that the main interaction site of SUMO-2 with DHPC micelles is at the N-terminal end of the hydrophobic cleft and the nearby loops. This location partially overlaps with the previously described protein interaction site of SUMO-2 at Q30 F31 I33. Of note, the loop of D62, and the corresponding loop in SUMO-1 were previously shown to interact with SUMO-conjugating enzyme Ubc9 [11, 15, 35] and the dipeptidyl peptidase DPP9 [53]. To prove that residues in the hydrophobic cleft and in the N-terminal loop indeed mediate membrane binding of SUMO-2, we introduced a series of mutations at residues Q30, F31, K32, I33, L42, Y46 within the hydrophobic cleft (SUMO-2 cleft) and additionally at H16, H36 and D62 in the N-terminal loop (SUMO-2 cleft + loop). Indeed, membrane binding of these mutants was decreased compared to SUMO-2 (Fig. 3f). Supporting our hypothesis that SUMO-2 interacts with lipid membranes for sorting into extracellular vesicles we found that the release of both mutants within extracellular vesicles was reduced compared to SUMO-2 (Fig. 3g, Supplementary Fig. S3b).

Having established SUMO modification as a novel sorting determinant for proteins into extracellular vesicles, we next investigated its relevance for the targeting of other proteins into extracellular vesicles. In neurodegenerative diseases, extracellular vesicles have been proposed as a potential carrier to disseminate misfolded proteins and thereby contribute to spreading of disease pathology [1]. A prototypic example is α-Synuclein, which is a major component of intracellular Lewy bodies (LBs) that neuropathologically define Parkinson’s disease (PD) and dementia with Lewy bodies (DLB) [62]. We have recently described two major sumoylation sites at K96 and K102 in α-Synuclein [38]. Therefore, we wondered if sumoylation of these sites might modulate extracellular release of α-Synuclein.

### α-Synuclein is localized in extracellular vesicles in vivo

It is not known whether α-Synuclein exists in extracellular vesicles in vivo or how it might be sorted to the extracellular vesicle pathway. To address these issues we first analysed whether α-Synuclein is present in extracellular vesicles in the human central nervous system (CNS). We prepared extracellular vesicles from lumbar cerebrospinal fluid by several centrifugation rounds including a final 100,000×*g* ultracentrifugation step (Supplementary Fig. S4a). Electron microscopy of the resulting pellet revealed 50–100 nm structures with morphological features of extracellular vesicles (Supplementary Fig. S4b). The pellet was enriched in the extracellular vesicle proteins Flotillin-2 and CD63 in addition to the Glutamate Receptors- 1, -2 and -3. The latter finding indicates that extracellular vesicles in the cerebrospinal fluid are at least partially derived from the CNS (Suppl. Fig. S4c). Microsomal proteins such as the endoplasmatic reticulum (ER) marker Calnexin and the trans-Golgi network (TGN) protein γ-Adaptin were absent, thus excluding organelle contamination of the extracellular vesicle preparation (data not shown). Of note, sumoylated proteins were detected in CSF-derived extracellular vesicles with a moderate enrichment of several proteins in this fraction (Supplementary Fig. S4d). On a sucrose gradient, Flotillin-2 was enriched in fractions with a density of 1.16–1.24 g/ml which is consistent with previously published results for Flotillin-positive extracellular vesicles [3] (Supplementary Fig. S4e). Western blot analysis of total cerebrospinal fluid and the corresponding 100,000×*g* pellet revealed the presence of α-Synuclein in the extracellular vesicle containing pellet (Supplementary Fig. S4f). Sucrose gradient centrifugation of CSF-derived extracellular vesicles followed by electrochemiluminescence assay detection of α-Synuclein revealed a flotation behaviour similar to the extracellular vesicle marker protein Flotillin-2 (Supplementary Fig. S4g). These findings establish that extracellular vesicle-associated α-Synuclein indeed exists in the CNS in vivo and might therefore play a key role in the pathology of the disease.

### α-Synuclein is predominantly localized in the lumen of extracellular vesicles

In line with previous results from studies of immortalized cell lines [16] we found by sucrose gradient and Western blot analysis that α-Synuclein is released with extracellular vesicles (electron micrograph, Supplementary Fig. S5a) from transiently transfected N2a cells and the oligodendroglial cell line Oli-neu (Supplementary Fig. S5b + c and data not shown). Overexpression of exogenous α-Synuclein might artificially lead to its extracellular vesicle-dependent release. However, the fact that α-Synuclein is present in the extracellular vesicle fraction in human cerebrospinal fluid argues in favour of extracellular vesicle-associated release even at physiological expression levels. In addition, we found by electrochemiluminescence assay analysis that endogenous α-Synuclein is released within extracellular vesicles at levels comparable to the extracellular vesicle marker protein Alix (Supplementary Fig. S5d).

Trypsin digestion of extracellular vesicle preparations revealed that α-Synuclein resides within the vesicles rather than being attached to the external membrane (Supplementary Fig. S5e + f). Trypsin digestion conditions were selected to ensure that Flotillin-2, a bona fide intraluminal extracellular vesicle protein, was protected from proteolysis. The silver gel shows degradation bands in the trypsin-treated 100,000×*g* pellet compared to the non-trypsinized control. Western blot analysis demonstrated that the content of Flotillin-2 and α-Synuclein in the extracellular vesicle pellet was unaltered by trypsin treatment, indicating that, as in the case of Flotillin, α-Synuclein is localized in the vesicle lumen (Supplementary Fig. S5e). In contrast, α-Synuclein was degraded to a similar extent as the intraluminal protein Alix when vesicles were trypsinized in the presence of 1 % Triton (Supplementary Fig. S5f).

### Sumoylation regulates extracellular vesicle release of α-Synuclein

The mechanism responsible for sorting of the cytosolic protein α-Synuclein into the extracellular vesicle pathway is unknown. To test whether sumoylation might regulate the release of α-Synuclein with extracellular vesicles, we transiently transfected N2a cells with Myc-α-Synuclein mutants which interfere with the protein’s sumoylation. Myc-α-Synuclein K96R K102R bears a double mutation (2KR) at two sumoylation sites which account for more than 50 % of SUMO conjugation. The D98A E104A double mutation (2AA) disrupts the consensus motif for recognition of adjacent SUMO receptor lysines [38]. As we have shown previously, neither the 2KR nor the 2AA mutation impairs ubiquitination of α-Synuclein while both reduce sumoylation to a similar extent [38].

**Fig. 4:**
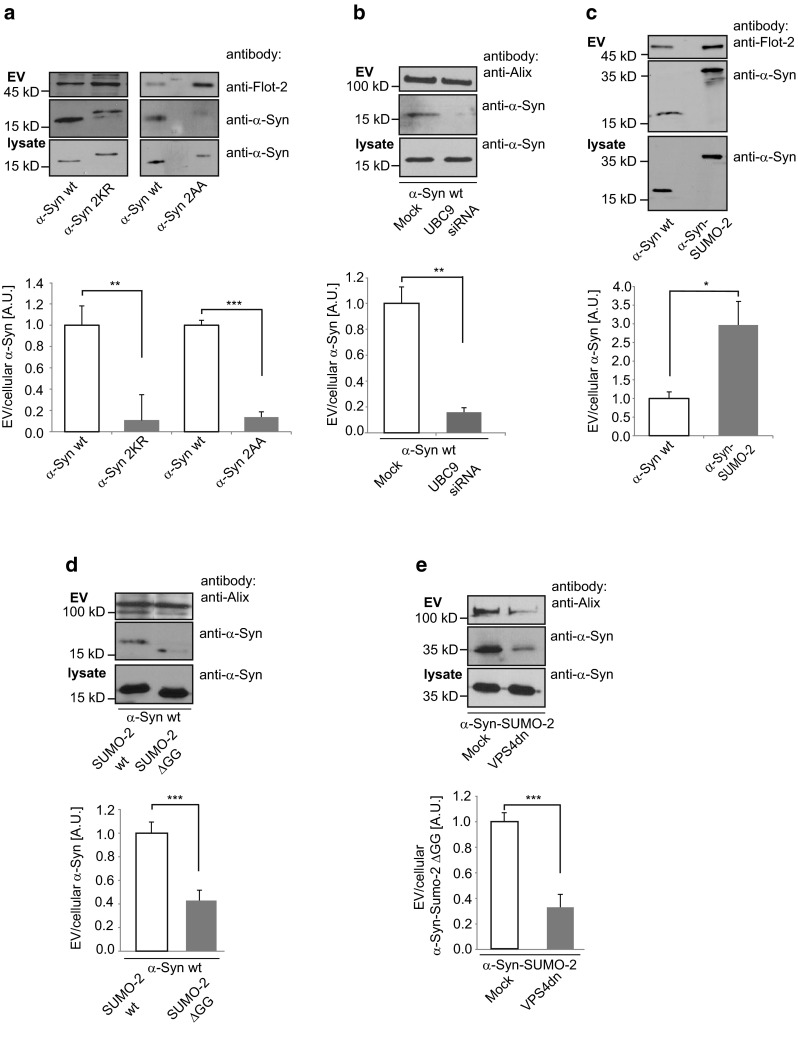
Sumoylation regulates release of α-Synuclein with extracellular vesicles. **a** Release of 2KR and 2AA mutants into extracellular vesicles from N2a cells (*n* = 6). **b** Human embryonic kidney (HEK) cells were treated with Ubc9 siRNA or mock treated 36 h prior to transfection with α-Synuclein wild type. The ratio of extracellular vesicle (EV) to cellular α-Synuclein (lys) was determined by Western blot analysis. Alix was blotted as a positive control for EV preparations. Results are given as means + SEM, **p < 0.005, two-side *t* test (*n* = 6). **c** α-Synuclein wild type and α-Synuclein-SUMO-2 were transiently transfected and extracellular vesicle release measured by Western blot (*n* = 8). **d** Co-transfection of α-Synuclein wild type with either SUMO-2 wild type or the conjugation-deficient SUMO-2 ∆GG mutant. Extracellular vesicle release of α-Synuclein was determined by Western blot analysis (*n* = 10). **e** α-Synuclein-SUMO-2 was co-transfected with a VPS4 dominant negative mutant E233Q and extracellular vesicle release of Myc-α-Synuclein-SUMO-2 was quantified. (*n* = 12). All results are given as mean + SEM; *p < 0.05; **p < 0.005, ***p < 0.0005. Student’s *t* test. The exosome blot membranes were additionally probed with an antibody against Flotillin-2 or Alix as an exosomal marker protein (*upper panel*). Total extracellular vesicle numbers were quantified by nanoparticle tracking analysis of the culture medium and showed no difference between wild type and mutant transfected cells (**a**, **c**, **d**) and a significant reduction of extracellular vesicle release in the case of VPS4dn co-transfection (**e**) (Table S1)

Both sumoylation-deficient mutants were significantly reduced in the extracellular vesicle fraction of N2a and adeno-associated virus (AAV) infected primary cortical neurons, supporting our hypothesis that sumoylation increases release within extracellular vesicles (Fig. 4a, Supplementary Fig. S6a). Consistent with these results, the silencing of the E2 conjugating enzyme Ubc9 by siRNA (Supplementary Fig. S6b) to prevent sumoylation, resulted in a strong decrease of α-Synuclein release within extracellular vesicles (Fig. 4b) [7].

Next, we generated a Myc-a-Synuclein-SUMO-2 fusion protein mimicking constitutive SUMO modification and harbouring the ∆GG mutation to prevent SUMO conjugation to other proteins.

In line with the notion that SUMO conjugation enhances α-Synuclein release within extracellular vesicles, Myc-α-Synuclein-SUMO-2 was highly enriched in the extracellular vesicle preparation (Fig. 4c, Supplementary Fig. S6c). In a similar fashion, enhancing SUMO modification of Myc-α-Synuclein wild type by co-expression with Myc-SUMO-2 wild type significantly increased the fraction of α-Synuclein in extracellular vesicles compared to co-expression of the conjugation-deficient SUMO mutant Myc-SUMO-2 ∆GG (Fig. 4d). No difference was observed for the extracellular vesicle marker proteins Flotillin-2 and Alix or the total extracellular particle numbers, indicating that overexpression of SUMO-2 does not increase the production of extracellular vesicles itself (Supplementary Fig. S7; Table S1).

We could not detect a marked enrichment of the sumoylated α-Synuclein band in extracellular vesicles on Western blots due to an isopeptidase activity in extracellular vesicles that resulted in rapid de-conjugation (Supplementary Fig. S8a). Therefore, we used a luciferase-based protein fragment complementation assay [12] which allowed direct measurement of α-Synuclein-SUMO-2 interaction. As demonstrated in Supplementary Fig. S8b the strong physical interaction between α-Synuclein and SUMO-2 suggests that sumoylated α-Synuclein is enriched in extracellular vesicles compared to total cell lysates (Supplementary Fig. S8b).

The release of sumoylated α-Synuclein within extracellular vesicles was ESCRT-dependent since co-expression of VPS4 dn strongly reduced the release of the constitutively “sumoylated” Myc-α-Synuclein-SUMO-2 fusion construct within extracellular vesicles (Fig. 4e). Similar to SUMO-2 and in contrast to PLP, α-Synuclein was absent from intraluminal vesicles in Rab5Q79L-transfected cells characterized by enlarged endosomes filled with intraluminal vesicles (Supplementary Fig. S9). This data indicate that α-Synuclein-positive extracellular vesicles might bud directly from the plasma membrane, although release via the multivesicular body pathway cannot be excluded.

### Release of α-Synuclein within extracellular vesicles is regulated by membrane binding

As demonstrated above, SUMO–ESCRT interaction involves the binding of SUMO to lipids. We therefore assumed that sumoylation regulates α-Synuclein release by modulating α-Synuclein binding to the formation sites of extracellular vesicles at the plasma membrane. Membrane binding of α-Synuclein was determined by mechanical disruption of the cells followed by 196,000×*g* ultracentrifugation of the postnuclear supernatant. The membrane-containing pellet and the membrane-free, cytosolic supernatant fraction were subjected to Western blot analysis. Membrane association of α-Synuclein has been described as a two-step process with binding of amino acids 3–25 followed by a conformational shift of residues 26–100 into a α-helical structure which cooperatively binds to the membrane [4, 8].

**Fig. 5:**
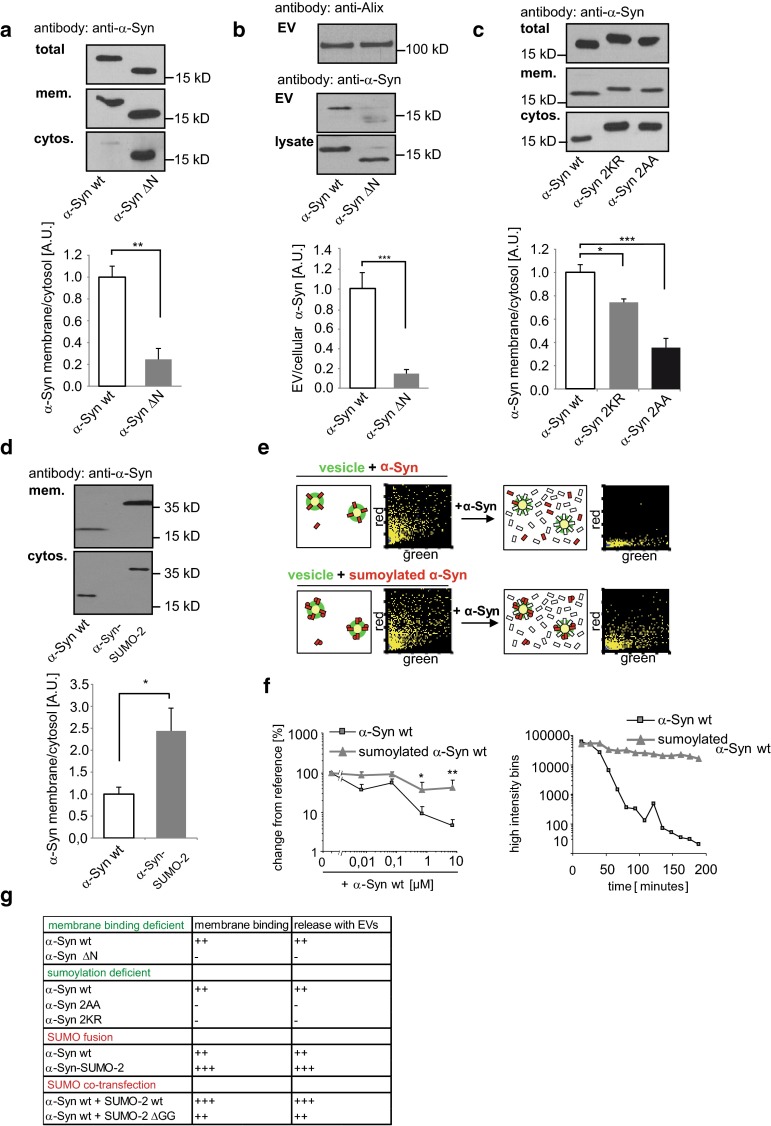
Membrane binding regulates release of α-Synuclein within extracellular vesicles. **a** N2a cells were transfected with wild-type or N-terminally truncated α-Synuclein (∆N). Cells were scraped and disrupted mechanically. The postnuclear supernatant was ultracentrifuged at 196,000×*g* for 30 min to separate the membrane-containing pellet and the cytosolic supernatant. Complete pellets and a proportion of the total cell lysate and the cytosol-containing supernatant were subjected to Western blot analysis with anti-α-Synuclein antibody to quantify the ratio of membrane associated α-Synuclein (*n* = 8). **b** Extracellular vesicles were prepared from the medium of N2a cells transfected with α-Synuclein wild type or ∆N and the ratio of extracellular vesicle to cell lysate protein was quantified upon Western blotting (*n* = 8). **c** Membrane pellets were prepared from cells after transfection with either α-Synuclein wild type (*n* = 12), the sumoylation-deficient mutant 2KR (*n* = 12) or the sumoylation consensus sequence mutant 2AA (*n* = 6). (**d**) Similar experiment as in (**c**) with transiently transfected α-Synuclein wild type and α-Synuclein-SUMO-2 (*n* = 6). **e** 2D fluorescence intensity histograms of SIFT recordings show binding of recombinant α-Synuclein (*red*) and sumoylated α-Synuclein (*red*) to DPPC lipid vesicles (*green*) (see also schematic drawing: α-Synuclein *red bar*; sumoylated α-Synuclein *red bar* with *dot*; DPPC lipid vesicles *green circle*). **f** In contrast to sumoylated α-Synuclein, non-sumoylated α-Synuclein is released from the lipid vesicles following addition of an ~1000-fold excess of unlabelled α-Synuclein (schematic drawing in **e** *white bar*). *Left* dose response curve for the effect of non-sumoylated α-Synuclein on the vesicle binding of sumoylated and non-sumoylated α-Synuclein (mean values + SEM normalized to reference (addition of buffer) of duplicate measurements of three parallel samples). *Right* time course of release of α-Synuclein and sumoylated α-Synuclein from lipid vesicles after addition of 7 µM unlabelled α-Synuclein in a representative experiment. **g** Summary of α-Synuclein membrane binding and release with extracellular vesicles. All results are given as mean + SEM; *p < 0.05; **p < 0.005, ***p < 0.0005. Student’s *t* test

Indeed, membrane binding of an N-terminal deletion construct of α-Synuclein lacking amino acids 2-19 (Myc-α-Synuclein ∆N) is significantly reduced [4, 34] (Fig. 5a, Supplementary Fig. S10a) and α-Synuclein ∆N was largely excluded from extracellular vesicles in contrast to wild-type α-Synuclein in transiently transfected N2a and Oli-neu cells (Fig. 5b and data not shown).

In line with our results above, we found that both SUMO-deficient α-Synuclein mutations, Myc-α-Synuclein 2KR and Myc-α-Synuclein 2AA significantly attenuated membrane binding of α-Synuclein compared to Myc-α-Synuclein wild type (Fig. 5c, Supplementary Fig. S10b).

In contrast, the Myc-α-Synuclein-SUMO-2 fusion construct was markedly enriched in the membrane pellet compared to Myc-α-Synuclein (Fig. 5d). Similar results were obtained in a fluorescence correlation spectroscopy (FCS) SIFTs assay [31], in which sumoylated recombinant α-Synuclein competed with non-sumoylated α-Synuclein for binding to dipalmitoyl-sn-glycero-3-phospho-choline (DPPC) vesicles (Fig. 5e + f). In contrast to sumoylated α-Synuclein, non-sumoylated α-Synuclein could be released from the lipid vesicles following the addition of a ~1000-fold excess of unlabelled α-Synuclein (Fig. 5f). Taken together, sumoylation of α-Synuclein promotes its binding to membranes, thereby increasing its release within extracellular vesicles (Fig. 5g).

## Discussion

Here, we show that sumoylation can serve as a sorting determinant for the release of proteins within extracellular vesicles. Our findings on SUMO-dependent sorting of GFP, APP and α-Synuclein into extracellular vesicles represent examples of this pathway.

After sucrose gradient flotation SUMO-2, APP and α-Synuclein were recovered at a density indicative of extracellular vesicles. For quantitative comparison of extracellular vesicle release, we preferred an ultracentrifugation protocol to sucrose gradient preparations. Therefore, one limitation of our study is a possible contamination of the ultracentrifugation pellet with other vesicles and protein aggregates [46, 67]. This especially concerns α-Synuclein which could be aggregated in the cell culture medium and co-sediment with extracellular vesicles during ultracentrifugation. However, the results from our trypsin digestion assay indicate that the vast majority of α-Synuclein recovered from the ultracentrifugation pellet is encapsulated in vesicles, rather than attached to the vesicular surface. Conflicting results had previously been reported for the localization of extracellular vesicle-associated α-Synuclein [12]. There, a substantial proportion of α-Synuclein in the extracellular vesicle preparation was accessible to trypsin digestion, indicating that α-Synuclein may be localized at the outer vesicle membrane. However, extracellular vesicles in that study were frozen after preparation and prior to trypsin digestion (Danzer, personal communication). In our assay, all vesicle preparations were digested immediately after preparation since freezing likely interferes with membrane integrity, making intravesicular protein accessible to trypsin. Under these experimental conditions, α-Synuclein was not degraded by trypsin and hence most likely localized within the vesicles.

Our results suggest that SUMO-2-dependent sorting to extracellular vesicles requires the ESCRT complex and is mediated by SUMO-2 interaction with phosphoinositols. Highest lipid-binding affinities were detected for SUMO-2 interaction with PI3P and PI(3,4,5)P3, indicating that both binding partners could serve as potential pathways to the ESCRT complex. Extracellular vesicles can either be derived from late endosomes/multivesicular bodies or shed from the plasma membrane. PI(3,4,5)P3 is predominantly localized at the plasma membrane in contrast to PI3P which is enriched at the endosomal membrane. Both lipids can interact with the ESCRT machinery. ESCRT-0 can be recruited to sites of intraluminal vesicle formation by binding of the Hrs FYVE domains to PI(3)P. Fusion of the cytosolic protein TyA with the PI(3,4,5)P3-binding domain of AKT protein kinase efficiently targets the protein to extracellular vesicle budding sites at the plasma membrane [58]. Based alone on the lipid-binding affinities of SUMO-2 for PI3P and PI(3,4,5)P3, it is not possible to distinguish between the multivesicular body and the plasma membrane shedding pathway for SUMO-2 release. We therefore overexpressed Rab5Q79L which blocks endosomal maturation and leads to the accumulation of intraluminal vesicles (ILVs) in enlarged endosomes. Using this method, we could neither detect SUMO-2 nor α-Synuclein in Rab5Q79L-induced endosomal ILVs. This suggests that SUMO-2 might bind to the plasma membrane for subsequent shedding into vesicles rather than being sorted into multivesicular endosome-derived ILVs. A spatial selectivity of SUMO-2 to PI(3,4,5)P3 binding at the plasma membrane may be caused by differences in the cholesterol to phospholipid ratio which is higher in the plasma membrane compared to endosomal membranes [72]. Interestingly, the presence of cholesterol enhances the binding of the tumour suppressor phosphatase and tensin homologue PTEN to a variety of different phosphoinositides [33], presumably by cholesterol-induced segregation of phosphoinositides, thereby reducing their electrostatic repulsion [33]. Sumoylation was previously described to mediate PTEN relocalization to the plasma membrane where it dephosphorylates PI(3,4,5)P3 [25, 32]. Of note, the release of PTEN within extracellular vesicles was reported recently [21]. Based on the rab5Q79L overexpression experiment alone, it cannot be excluded that small amounts of SUMO-2 bud into multivesicular endosomes which are not detected by microscopy. In addition, rab5Q79L overexpression may not inhibit the maturation of all endosomes to multivesicular endosomes. Although vesicles within rab5Q79L endosomes are positive for a set of ILV marker proteins and their morphology resembles that of ILVs within multivesicular bodies derived from late endosomes, we cannot rule out that there are slight differences between those vesicles trapped within rab5Q79L endosomes and those present in multivesicular endosomes. We can therefore not exclude that SUMO-2 is released by the multivesicular endosome/exosome pathway. Especially, since both types of extracellular vesicles cannot be separated by ultracentrifugation and sucrose gradient centrifugation protocols or based on specific marker proteins, further experimentation is clearly required to ultimately distinguish between both pathways.

Taken together, our data on GFP-SUMO-2, α-Synuclein and APP strongly support the concept of a SUMO modification as a novel sorting signal to extracellular vesicles which is ESCRT-dependent. Targeting of proteins to the vesicle formation site may be achieved by interaction with phosphorylated inositols, possibly at the plasma membrane.

A prion-like transmission of α-Synuclein aggregates including intercellular spreading and templating of further pathological aggregate formation in recipient cells had been proposed recently [6, 57]. This hypothesis is mainly based on the temporo-spatial spreading of α-Synuclein pathology in Parkinson’s disease [10] and further supported by the observation of α-Synuclein aggregates in embryonic midbrain neurons which had been transplanted into Parkinson’s disease patients’ brains [36, 43]. Further evidence stemmed from murine in vivo and in vitro models, where the uptake of recombinant α-Synuclein fibrils in neurons was detected, followed by the induction of α-Synuclein aggregation [12, 16, 44, 45, 49, 50, 74]. Several reports have indeed shown a-Synuclein associated with extracellular vesicles [17, 69] and recently we provided evidence for the exosome-mediated propagation of oligomeric α-Synuclein in vitro [12].

However, in the absence of a conventional secretion signal in α-Synuclein, the mechanisms which lead to its extracellular release remained elusive so far. Our results show for the first time that α-Synuclein is present in extracellular vesicles in the CNS in vivo and shed light on the molecular mechanisms which direct α-Synuclein into vesicles. Our findings are thus of highest relevance for the understanding of Parkinson’s disease pathogenesis and progression at the molecular level. Therefore, our results may have important consequences for the development of novel therapeutic strategies to treat Parkinson’s disease. In summary, our findings also assign SUMO modification of proteins a previously unknown cell biological function.

## Electronic supplementary material

Below is the link to the electronic supplementary material.

Supplementary material 1 (TIFF 19589 kb)

Supplementary material 2 (TIFF 12909 kb)

Supplementary material 3 (TIFF 20209 kb)

Supplementary material 4 (TIFF 9416 kb)

Supplementary material 5 (TIFF 25524 kb)

Supplementary material 6 (PNG 286 kb)

Supplementary material 7 (PNG 81 kb)

Supplementary material 8 (TIFF 7883 kb)

Supplementary material 9 (TIFF 13906 kb)

Supplementary material 10 (TIFF 3148 kb)

Supplementary material 11 (DOCX 48 kb)
