# Supplementary figures and images for: Extracellular vesicle sorting of α-Synuclein is regulated by sumoylation
Source: Acta Neuropathol. 2015 Mar 17;129(5):695–713. doi: 10.1007/s00401-015-1408-1 (PMC4405286; doi:10.1007/s00401-015-1408-1)

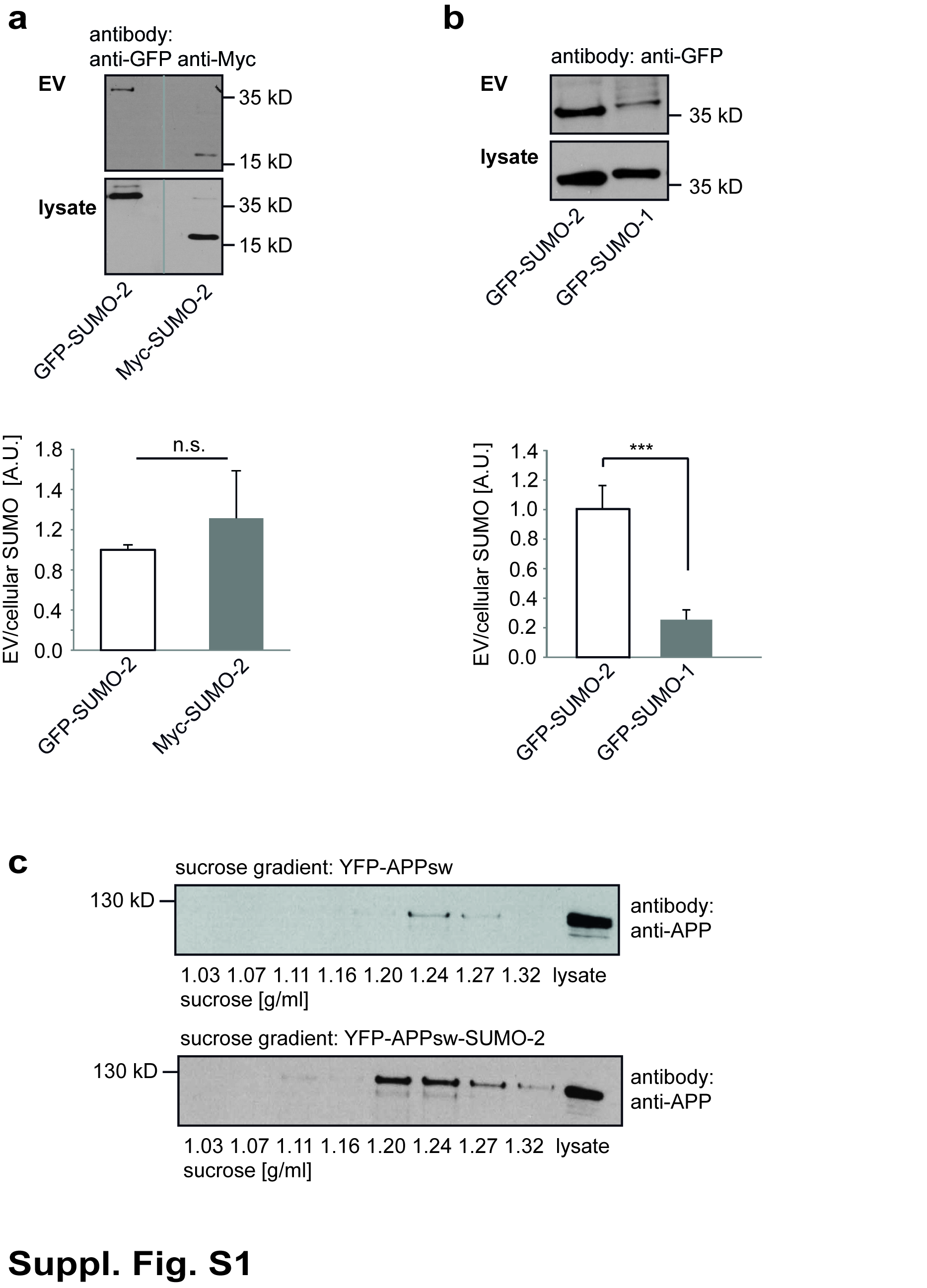

Supplement: Supplementary file 1 — Supplementary material 1 (TIFF 19589 kb) [file 401_2015_1408_MOESM1_ESM.tif]

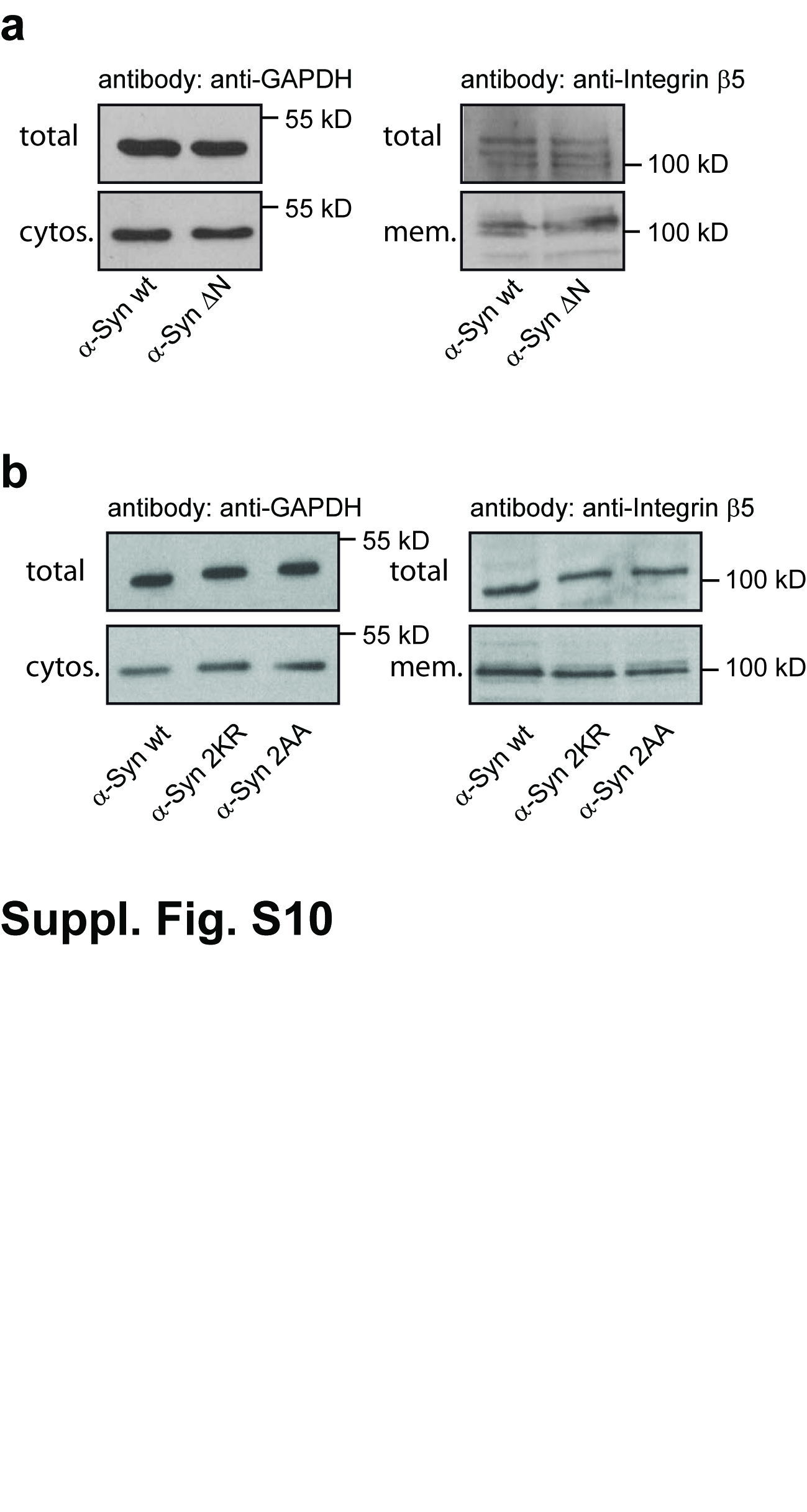

Supplement: Supplementary file 2 — Supplementary material 2 (TIFF 12909 kb) [file 401_2015_1408_MOESM2_ESM.tif]

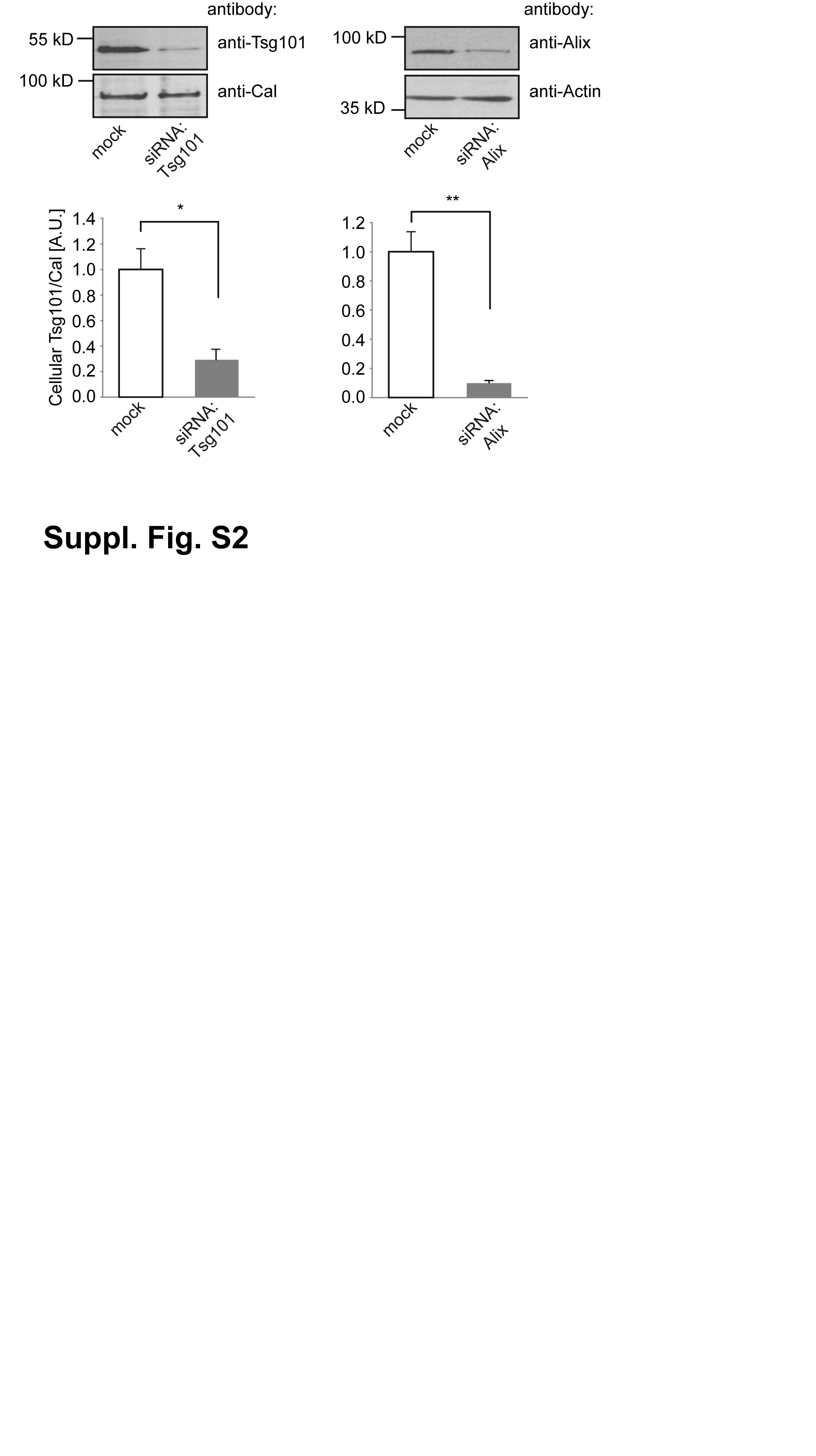

Supplement: Supplementary file 3 — Supplementary material 3 (TIFF 20209 kb) [file 401_2015_1408_MOESM3_ESM.tif]

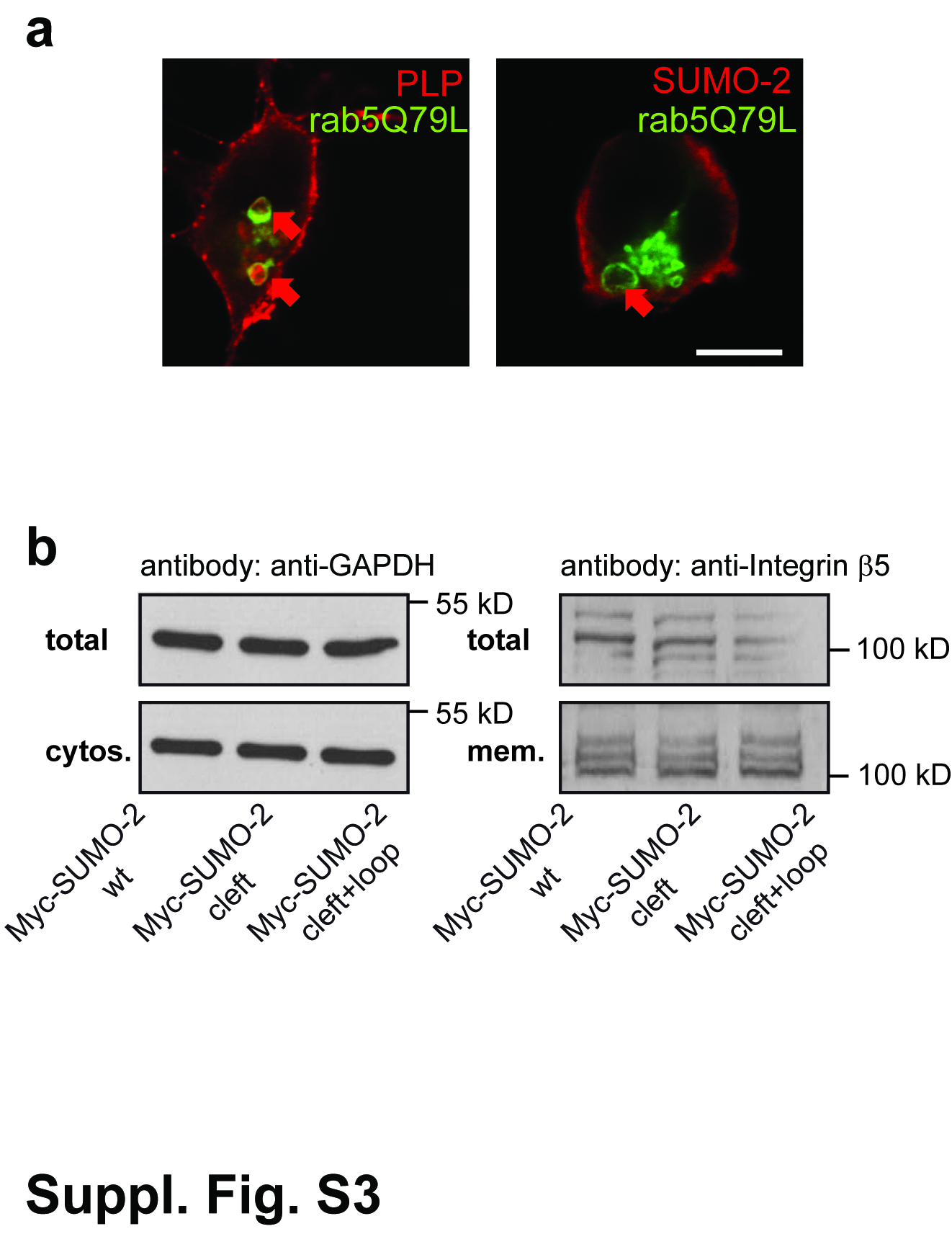

Supplement: Supplementary file 4 — Supplementary material 4 (TIFF 9416 kb) [file 401_2015_1408_MOESM4_ESM.tif]

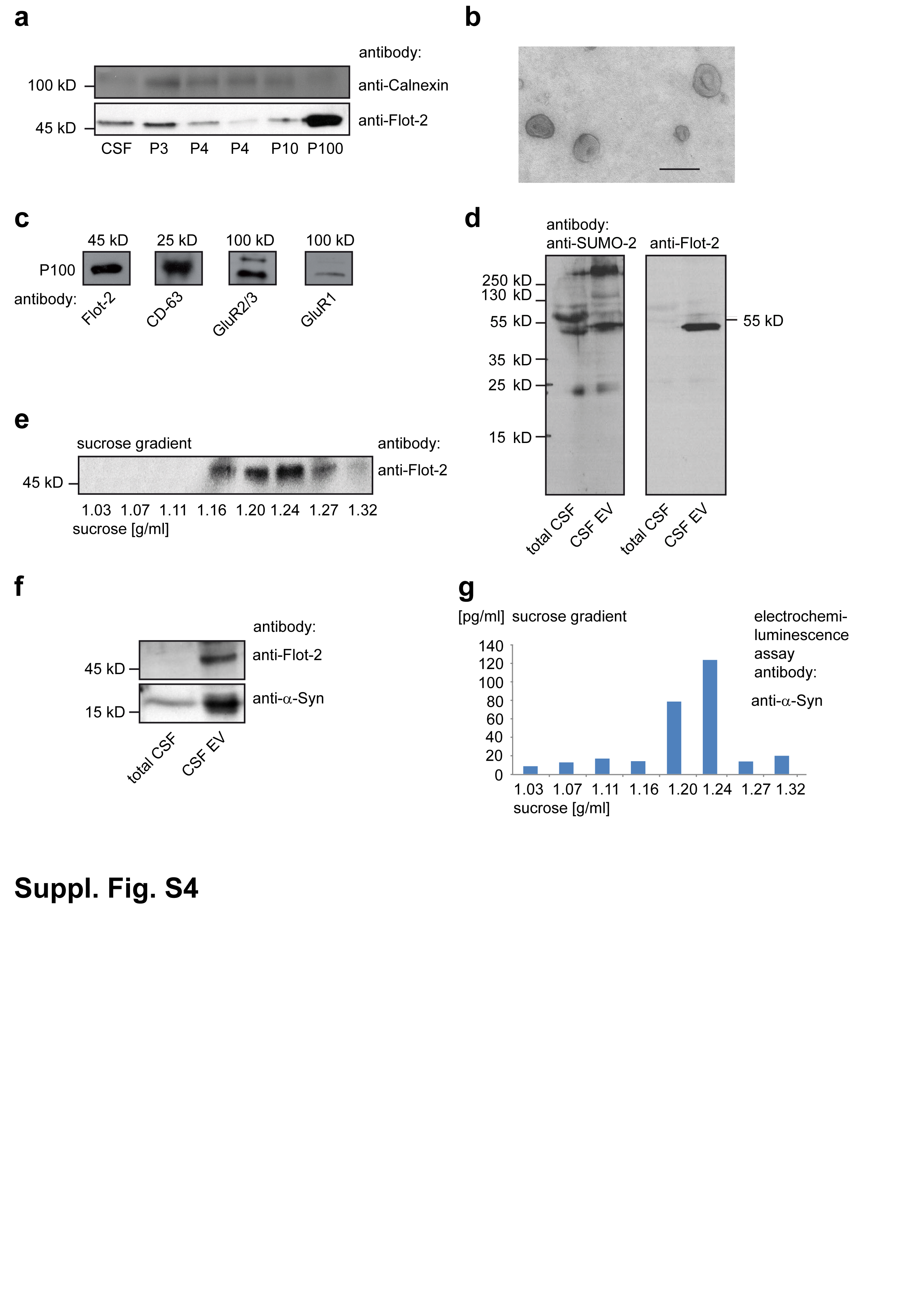

Supplement: Supplementary file 5 — Supplementary material 5 (TIFF 25524 kb) [file 401_2015_1408_MOESM5_ESM.tif]

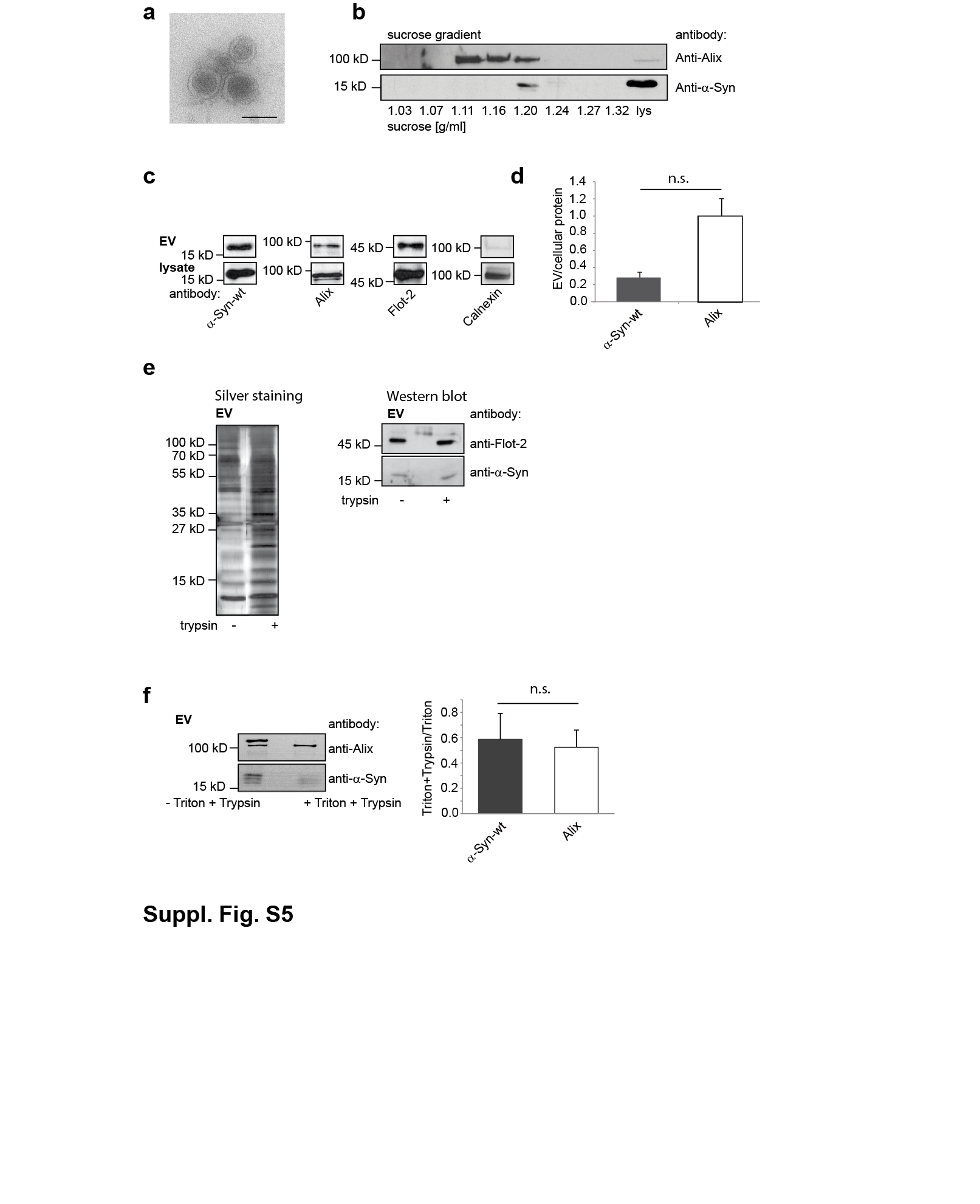

Supplement: Supplementary file 6 — Supplementary material 6 (PNG 286 kb) [file 401_2015_1408_MOESM6_ESM.png]

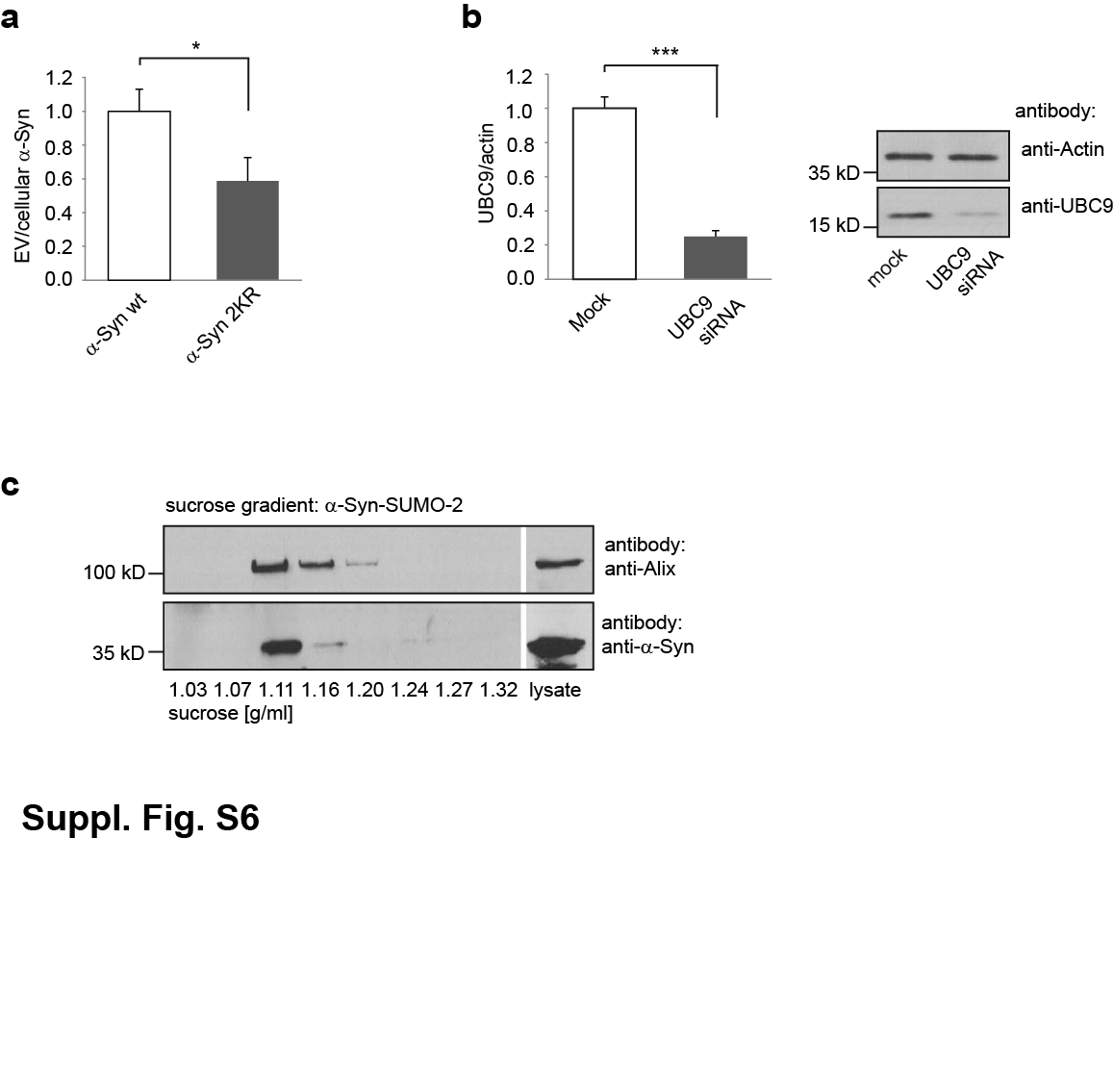

Supplement: Supplementary file 7 — Supplementary material 7 (PNG 81 kb) [file 401_2015_1408_MOESM7_ESM.png]

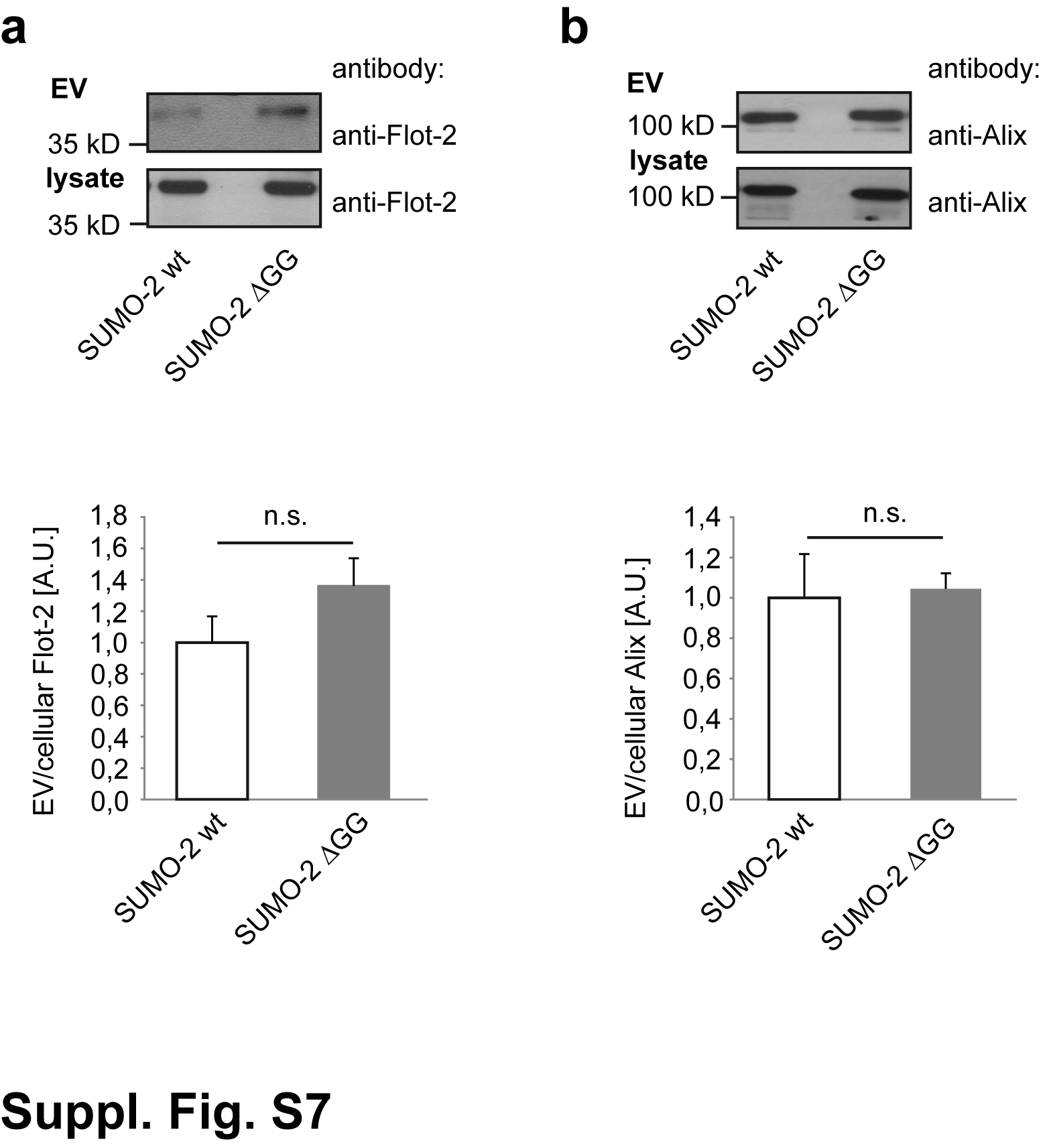

Supplement: Supplementary file 8 — Supplementary material 8 (TIFF 7883 kb) [file 401_2015_1408_MOESM8_ESM.tif]

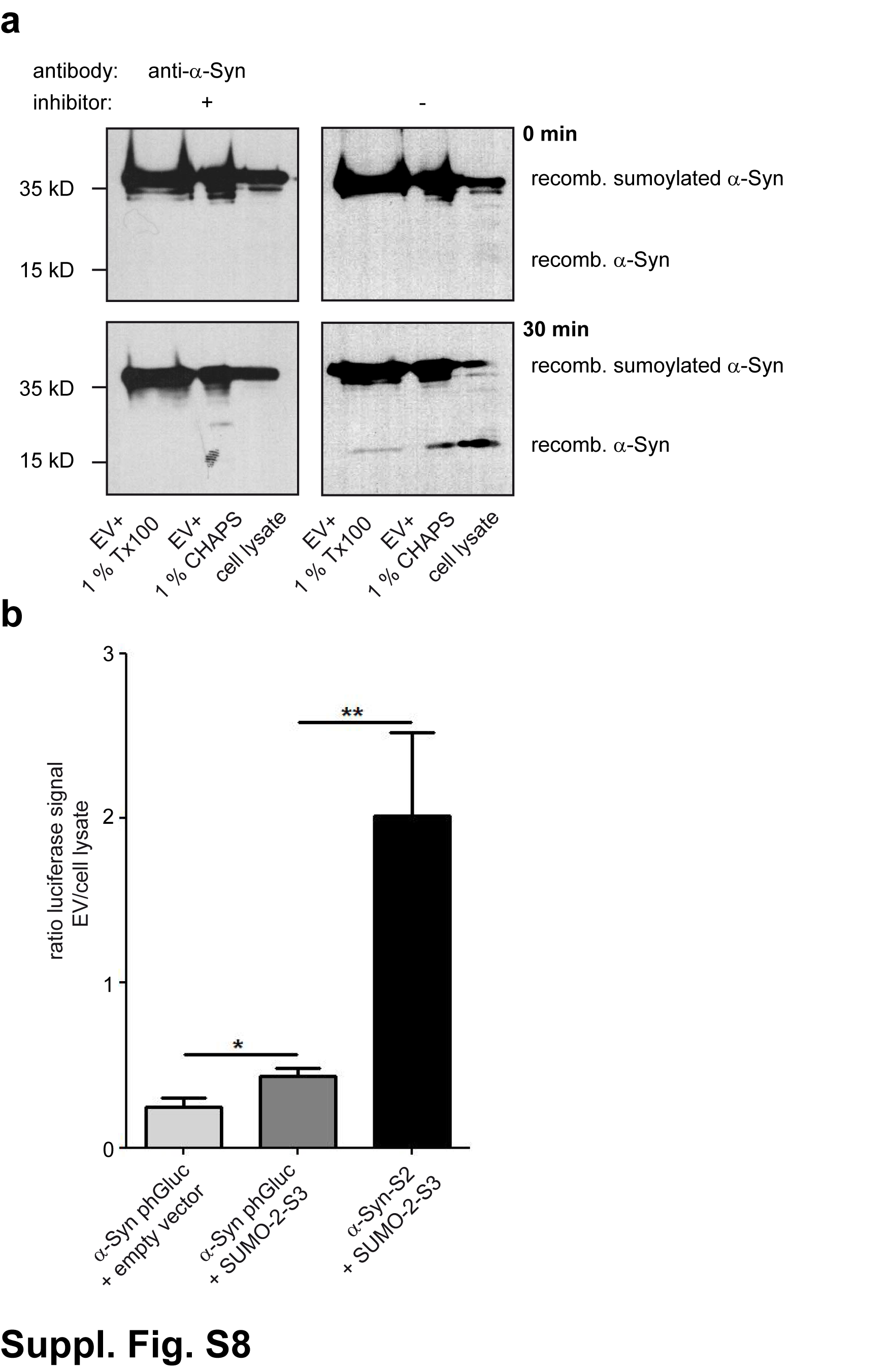

Supplement: Supplementary file 9 — Supplementary material 9 (TIFF 13906 kb) [file 401_2015_1408_MOESM9_ESM.tif]

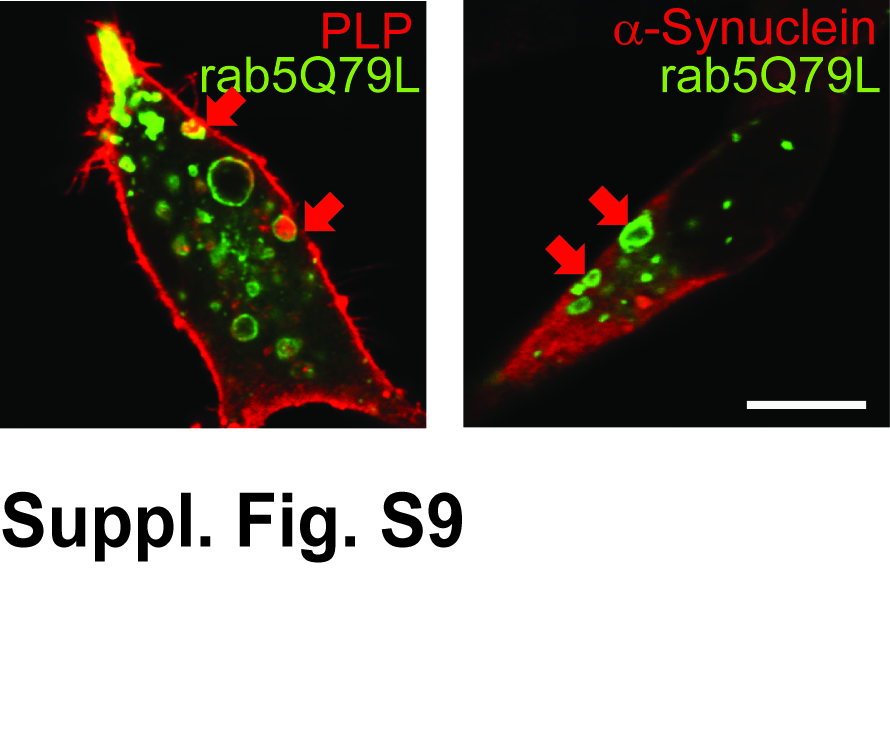

Supplement: Supplementary file 10 — Supplementary material 10 (TIFF 3148 kb) [file 401_2015_1408_MOESM10_ESM.tif]
