# Supplementary material for: Extracellular vesicle sorting of α-Synuclein is regulated by sumoylation
Source: Acta Neuropathol. 2015 Mar 17;129(5):695–713. doi: 10.1007/s00401-015-1408-1 (PMC4405286; doi:10.1007/s00401-015-1408-1)
Supplement: Supplementary file 11 — Supplementary material 11 (DOCX 48 kb) [file 401_2015_1408_MOESM11_ESM.docx]

**Exosomal sorting of α-Synuclein for extracellular release is regulated by sumoylation**

**Authors:** Marcel Kunadt ^1,2,3^, Katrin Eckermann ^2,4^, Katrin Strauss ^1,2,3^, Surya Rai ^1,3^, Sebastian Kügler ^2,4^, Anne Stündl ^1^, Lisandro Falomir Lockhart ^5^, Martin Schwalbe ^6^, Petranka Krumova ^4^, Luis M. A. Oliveira ^5^, Mathias Bähr ^2,4^, Wiebke Möbius ^2,3^, Albert C. Ludolph ^14^, Johannes Levin ^7^, Armin Giese ^8^, Niels Kruse ^9^, Brit Mollenhauer ^9,10^, Ruth Geiss-Friedlander ^11^, Axel Freischmidt ^12^, Marisa Feiler ^12^, Karin M. Danzer ^12^, Markus Zweckstetter ^2, 6, 13^, Thomas M. Jovin ^2,5^, Mikael Simons ^2,3,4^, Jochen H. Weishaupt ^2,4,14,#^, Anja Schneider ^1,2,3,13*,#^

Supplementary Material:

Figures S1-S10

Table S1

**Figure legends S1-S10**

**Fig. S1 SUMO-2 is released with extracellular vesicles**. (a) N2a cells were transiently transfected with SUMO-2 either fused to GFP or Myc. Both proteins are found in the extracellular vesicle fraction to a similar extent (n=6). (Please note that the EV and lysate blots were cut for incubation with either anti-GFP or anti-Myc antibody. Exposure times were identical). (b) N2a cells were transiently transfected with conjugation-deficient SUMO-2 or SUMO-1 (n=12). As a negative control, we quantified release of transiently transfected EYFP with extracellular vesicles (EV) with release of SUMO-2 normalised to 1. EV/lysate ratio of EYPF was 0.026 (n=3, SEM=0.0037, p=0.0479; data not shown).

Western blots of the extracellular vesicle preparation and lysates were quantified and the EV/lysate ratio of SUMO-2 was arbitrarily set to 1.0. Results are given as mean+SEM, *** p<0.0005, 2-side t-test. (c) A discontinous sucrose step gradient (0.25-2.5M) was loaded onto the 100,000 g extracellular vesicle pellet of cells either transfected with APPsw (top panel) or APP-SUMO-2 (bottom panel). Fractions were analyzed by Western blotting with anti-APP antibody.

**Fig. S2**  **Down-regulation of Tsg101 (left) and Alix (right) with siRNA.** Efficiency of siRNA-mediated down-regulation was quantified by Western blotting of cell lysates with antibodies against Tsg101 and calnexin as loading control or Alix and Actin as loading control, respectively. The ratio Tsg101/Calnexin or Alix/Actin was quantified. Results are given as mean+SEM, student’s 2-side t-test (n=3 for Tsg101, n=6 for Alix), ** p<0.005, * p<0.05.

**Fig. S3 (a) Subcellular distribution of SUMO-2.** N2a cells were co-transfected with Rab5Q79L-GFP (green) and PLP-Myc (red) (left) or Rab5Q79L-GFP (green) and SUMO-2-Myc (red) (right) for 24 hours. Images were taken by laser scanning confocal microscopy. Localization of PLP within Rab5Q79L endosomes is indicated by red arrows. No localization of SUMO-2 within Rab5Q79L endosomes was observed (n= 20 endosomes per condition). Scale bar, 10 μm. **(b) Fractionation controls for membrane preparation experiments.** Total cell lysates as well as membrane and cytosol fractions were subjected to Western blot analysis to determine the distribution of SUMO-2 in both fractions (Fig.3). These blots were re-probed with antibodies directed against GAPDH as a cytosol marker and against Integrin β5 as a membrane marker. Left panel: Total and cytosol (cytos.) fractions of Myc-SUMO-2, Myc-SUMO-2 cleft and Myc-SUMO-2 cleft+loop transfected cells probed with GAPDH antibody, right panel: total and membrane (mem.) fraction of the same experiment probed with an anti-Integrin β5 antibody.

**Fig. S4 Characterization of extracellular vesicles in cerebrospinal fluid.** (a) Cerebrospinal fluid was centrifuged at 3,500×*g* (pellet=P3), the resulting supernatant was centrifuged twice at 4,500 ×*g* (pellets P4), the respective supernatants were subjected to centrifugation at 10,000×*g* (P10) and 100,000×*g* (P100). Pellets were analyzed by Western blotting with antibodies against Calnexin (negative control) and Flotillin-2 (positive control). (b) 100,000×*g* pellets were prepared from cerebrospinal fluid and negatively stained with 1% uranyl acetate. Scale bar 100 nm. (c) 100,000×*g* pellets were analyzed by Western blot against various microsomal and extracellular vesicle marker proteins. (d) Detection of sumoylated proteins in CSF EVs. Two different CSF samples were pooled. EVs were prepared by ultracentrifugation and unfractionated CSF (left) as well as the EV fraction (right) were subjected to Western blotting and probed with an antibody against SUMO-2 (left panel) and Flotillin-2 (right panel). (e) The 100,000×*g* pellet was subjected to a discontinuous sucrose gradient (0.25-2.5M) and the fractions were analyzed by Western blotting for the presence of Flotilllin-2. (f) Extracellular vesicles were prepared from 5 ml cerebrospinal fluid. 20 μl of total cerebrospinal fluid and the resulting extracellular vesicles were subjected to Western blotting with an antibody against α-Synuclein and Flotillin-2 as an extracellular vesicle marker. One representative blot out of 3 different patient samples is shown. (g) The 100,000xg pellet from a Parkinson dementia CSF sample was loaded on a discontinuous sucrose gradient (0.25-2.5M) and α-Synuclein content of each fraction was quantified by an electrochemiluminescence assay.

**Fig. S5 Characterization of α-Synuclein within extracellular vesicles derived from N2a cells.** (a) N2a cells were transfected with a plasmid to express α-Synuclein wild-type. Extracellular vesicles were prepared from culture medium and processed for electron microscopy (scale bar 100 nm). (b) A discontinous sucrose step gradient (0.25-2.5M) was loaded onto the 100,000 g extracellular vesicle pellet. Fractions were analyzed by Western blotting with anti-α-Synuclein antibody. (c) Cell lysates and extracellular vesicle fractions were probed with different antibodies (Calnexin: marker of microsomal contamination, Alix and Flotillin-2: extracellular vesicle proteins, α-Synuclein). While Calnexin was readily detectable in cell lysates, it was absent in the extracellular vesicle fraction. (d) EV release of endogenous a-Synuclein from HEK cells was measured by electrochemiluminescence assay, the ratio of Alix in EV fractions and cell lysates was determined by Western Blot analysis. The ratio of a-Synuclein and Alix (EVs/lysate) was calculated and normalized to 1.0 for Alix. Results are shown in the histograph (results are shown as mean, +SEM, n=3, no significant difference, 2-sided student’s t-test). (e) 100,000 g pellets were resuspended in PBS and half of the preparation was digested with trypsin at a final concentration of 0.0125% for 5 min at 37°C (left lane). As a control, the other half of the extracellular vesicle preparation was incubated in PBS at 37° C (right lane). Trypsination was stopped by adding sample buffer and heating to 95 °C. Trypsination efficiancy was controlled by SDS PAGE and silver staining. Right panel: Western blot analysis of trypsinated (left lane) and not-trypsinated (right lane) extracellular vesicles. Membranes were probed with antibodies against Fotillin-2 and α-Synuclein. (f) Similar experiment as in (e). 100.000 g pellets were resuspended in PBS and 0.00084% trypsin and incubated for 3 min at 37°C, either in the absence (left lane) or presence (right lane) of 1% Triton X100 followed by a 3 min incubation at 37° C. Trypsination was stopped by adding sample buffer and heating to 95°C. Samples were subjected to Western blot analysis with antibodies against α-Synuclein and Alix. The degradation of both proteins by trypsination was quantified by calculating the ratio of protein under trypsin plus triton condition versus trypsin without triton condition. No significant difference was obtained between α-Synuclein and Alix degradation.

**Fig. S6 Primary cortical neuron cultures were infected with adeno-associated virus to express either α-Synuclein wild-type or the sumoylation-deficient mutant α-Synuclein 2KR.** (a) Extracellular vesicles were prepared from culture medium and α-Synuclein was quantified in extracellular vesicle fractions and cell lysates by an electrochemiluminescence assay. The ratio of α-Synuclein within extracellular vesicles versus cellular α-Synuclein is shown in the histograph (results given as mean+SEM; wild-type normalized to 1; * p<0.05, 2-side t-test (n= 15)). (b) “Down-regulation of Ubc9 with siRNA. Efficiency of siRNA-mediated down-regulation was quantified by Western blotting of cell lysates with antibodies against Ubc9 and actin as loading control. The ratio Ubc9/Actin was quantified. Results are given as mean+SEM, student’s 2-side t-test (n=8), *** p<0.0005.”

**Fig. S7 Expression of SUMO-2 does not increase release of extracellular vesicles per se.** Cells were co-transfected with α-Synuclein wild-type with either SUMO-2 wild-type or the conjugation-deficient SUMO-2 mutant ΔGG. (a) Extracellular vesicle release of endogenous Flotillin-2 and (b) Alix was quantified by Western blot analysis (ratio of EV protein/protein in cell lysate). Results are given as mean +SEM; n.s.=not significant, student‘s 2-side t-test (n=6).

**Fig. S8** (a) **Extracellular vesicles contain desumoylase activity.** Extracellular vesicles were prepared from N2a cells and lysed with either 1% Triton or 1% CHAPS. N2a cell lysates were prepared with 1% CHAPS buffer. Recombinant sumoylated α-Synuclein was incubated for 0 min (top panel) or 30 min at 37 °C (bottom panel) with extracellular vesicles or cell lysates in the presence (left panel) or absence (right panel) of N-ethylmaleimide which inhibits desumoylases. Samples were subjected to Western blot analysis with an antibody against α-Synuclein. One representative experiment is shown (out of n=3). (b) **Enrichment of sumoylated α-Synuclein in extracellular vesicles.** HEK293 cells were transfected with the indicated constructs. Luciferase activity was measured in extracellular vesicle (EV) fractions and total cell lysates (as described in material and methods). The EV/cell lysate luciferase activity ratio increased when α-Synuclein fused to full length gaussia luciferase (α-Syn phGluc) was co-expressed with SUMO-2, confirming that α-Synuclein is targeted to extracellular vesicles when sumoylated to a higher degree. When C- or N-terminal fragments of split phGluc were fused to α-Synuclein (α-Syn-S2) or SUMO-2 (SUMO-2-S3), only α-Synuclein modified by SUMO-2 resulted in luciferase signal (data not shown). This signal was further enriched in extracellular vesicle fractions relative to total cell lysates, indicating that sumoylated α-Synuclein is present and enriched in extracellular vesicles. (results are given as mean + SEM; student’s 2-side t-test, * p ≤ 0.05; ** p ≤ 0.01 (n = 3)).

**Fig. S9 Subcellular distribution of α-Synuclein.** Oli-neu cells were co-transfected with Rab5Q79L-GFP (green) and PLP-Myc (red) (left) or Rab5Q79L-GFP (green) and α-Synuclein-Myc (red) (right) for 24 hours. No localization of α-Synuclein or PLP within Rab5Q79L endosomes was observed (n= 20 endosomes per condition). Scale bar, 10 μm.

Fig. S10 **Fractionation controls for membrane preparation experiments.** Total cell lysates as well as membrane and cytosol fractions were subjected to Western blot analysis to determine the distribution of α-Synuclein in both fractions (Fig.5). The blots were re-probed with antibodies directed against GAPDH as a cytosol marker and against Integrin β5 as a membrane marker. (a) Left panel: Total and cytosol (cytos.) fraction of α-Synuclein wt and α-Synuclein ΔN transfected cells probed with GAPDH antibody, right panel: total and membrane (mem.) fraction of α-Synuclein wt and α-Synuclein ΔN transfected cells probed with an anti-Integrin β5 antibody. (b) Left panel: Total and cytosol (cytos.) fraction of α-Synuclein wt, 2KR and 2AA transfected cells probed with GAPDH antibody as positive control, right panel: total and membrane (mem.) fraction of α-Synuclein wt, 2KR and 2AA transfected cells probed with an anti-Integrin β5 antibody as positive control.

**Table S1**

|  | **mean particle concentration adjusted to cell number** | **SEM** |
| --- | --- | --- |
|  |  |  |
| **Myc-SUMO-2 wt/α-Syn wt** | 2.52E+07 | 1.67E+06 |
| **Myc-SUMO 2ΔGG/α-Syn wt** | 2.22E+07 | 2.31E+06 |
| **n** | 8 |  |
| **t-test** | 0.16738 |  |
|  |  |  |
| **α-Syn wt** | 2.24E+07 | 2.98E+06 |
| **α-Syn-SUMO-2** | 2.71E+07 | 4.15E+06 |
| **n** | 7 |  |
| **t-test** | 0.06368 |  |
|  |  |  |
| **α-Syn wt** | 2.16E+07 | 1.94E+06 |
| **α-Syn 2KR** | 2.07E+07 | 1.76E+06 |
| **α-Syn AA** | 2.36E+07 | 4.95E+06 |
| **n** | 6 |  |
| **t-test (wt/2KR)** | 0.55184 |  |
| **t-test (wt/2AA)** | 0.17285 |  |
|  |  |  |
| **α-Syn wt** | 3.33E+07 | 3.78E+06 |
| **α-Syn wt/VPS4dn co-transfection** | 2.50E+07 | 5.12E+06 |
| **n** | 7 |  |
| **t-test** | 0.000054 |  |
|  |  |  |
| **α-Syn-SUMO-2** | 5.10E+07 | 1.40E+05 |
| **α-Syn-SUMO-2 /VPS4dn** | 4.07E+07 | 5.35E+06 |
| **n** | 8 |  |
| **t-test** | 0.00281561 |  |
|  |  |  |
| **YFP-APPsw** | 3.30E+07 | 3.50E+05 |
| **YFP-APPsw-SUMO-2** | 3.85E+07 | 2.96E+05 |
| **n** | 8 |  |
| **t-test** | 0.104734285 |  |
|  |  |  |
| **YFP-APPsw/SUMO-2-wt** | 4.60E+07 | 3.47E+06 |
| **YFP-APPsw/SUMO-2-ΔGG** | 4.02E+07 | 1.82E+06 |
| **n** | 8 |  |
| **t-test** | 0.092198482 |  |
|  |  |  |
